# Space–time clustering of childhood cancers: a systematic review and pooled analysis

**DOI:** 10.1007/s10654-018-0456-y

**Published:** 2018-11-16

**Authors:** Christian Kreis, Eliane Doessegger, Judith E. Lupatsch, Ben D. Spycher

**Affiliations:** Institute of Social and Preventive Medicine (ISPM), University of Bern, Mittelstrasse 43, 3012 Bern, Switzerland

**Keywords:** Childhood cancer, Childhood leukaemia, Aetiology, Cancer registry, Cluster analysis, Meta-analysis

## Abstract

The aetiology of childhood cancers remains largely unknown. Space–time clustering of cases might imply an aetiological role of infections. We aimed to review the evidence of space–time clustering of specific childhood cancers. We searched Medline and Embase for population-based studies that covered a pre-defined study area, included cases under 20 years of age and were published before July 2016. We extracted all space–time clustering tests and calculated the proportion of positive tests per diagnostic group. In a pooled analysis, we performed a Knox test of the number of pairs of cases close to each other in time and space pooled across studies. 70 studies met our eligibility criteria, 32 of which reported Knox tests. For leukaemia, the proportion of positive tests was higher than expected by chance at both time of diagnosis (26%) and birth (11%). The pooled analysis showed strong evidence of clustering at diagnosis for children aged 0–5 years for a spatial and temporal lag of 5 km and 6 months, respectively (*p* < 0.001). The evidence was mixed for lymphoma and tumours of the central nervous system. The current study suggests that leukaemia cases cluster in space–time due to an aetiological factor affecting children under 5 years of age. The observed pattern of clustering of young children close to time of diagnosis is compatible with Greaves’ delayed-infections-hypothesis.

**Electronic supplementary material:**

The online version of this article (10.1007/s10654-018-0456-y) contains supplementary material, which is available to authorized users.

## Introduction

Cancers in childhood are rare and their aetiology remains largely unknown. There is some evidence that infections might be involved in the aetiology of certain childhood cancers. Epstein–Barr Virus (EBV) is known to be associated with Burkitt lymphoma and Hodgkin’s lymophoma [1, 2]. It has also been hypothesized that childhood leukaemia could have an infectious aetiology [3–5]. If this were the case, incidence might vary in time and space reflecting the circulation dynamics of the causative infectious agent. Specifically, cases may tend to occur in closer spatial and temporal proximity of one another than would be expected if their locations and times were independent [6, 7]. Evidence of such space–time clustering of cases could thus provide important clues about a putative role of infections. Studies of space–time clustering of childhood cancers emerged during the early 1960s, fuelled by recurrent reports of local clusters or “micro-epidemics” of childhood leukaemia and the accumulating evidence that viruses could cause leukaemia in certain animals [8]. Historically, it was these early studies of the spatial distribution of childhood cancers that spurred the development of statistical tests for space–time clustering of diseases (see Box in online supplementary material).

If childhood cancers follow a consistent pattern of space–time clustering due to some aetiological factor that manifests itself across different countries and time periods, a systematic review of clustering studies should pick this up. Furthermore, a meta-analytical approach summarising the evidence of clustering for the time points of birth or diagnosis, for different age groups and at different spatial and temporal scales might provide hints about windows of susceptibility and possibly even the likely range of putative aetiological agents. However, despite numerous studies carried out since the pioneering studies and a number of reviews, the evidence in support of space–time clustering of childhood cancers is equivocal and inferences about aetiology and, specifically, a putative role of infections remain elusive.

Previous literature reviews were all narrative reviews that did not attempt any quantitative evidence synthesis. Early reviews of studies of childhood leukaemia were either inconclusive [8, 9] or deemed the evidence unconvincing [10]. Subsequent reviews observed weak space–time clustering [11, 12], notably of acute lymphoblastic leukaemia (ALL) in young children [11]. Finally, a review of 33 studies of childhood leukaemia published up to 2002 concluded that the evidence supported space–time clustering for time of diagnosis and probably also for time of birth [13]. Except for Burkitt lymphoma [10, 12], no consistent evidence of space–time clustering was observed for childhood lymphomas, nor for central nervous system (CNS) tumours or rarer childhood cancers [12].

Furthermore, previous reviews failed to appraise the quality of the data and methodology of the included studies. Earlier space–time clustering studies often retrieved case data from death certificates and used combinatorial statistics to assess spatio-temporal patterns. Later analyses were based on medical records or specialized cancer registries and used formal statistical tests specifically developed to detect clustering of cases. Also, many clustering studies relied on imprecise address information geocoded to administrative areas or employed numerous statistical tests without correcting for multiple testing. Finally, few studies adjusted for uneven shifts in the background population, which risks producing spurious evidence of space–time clustering in statistical tests of case-only data [14].

We systematically searched the literature for original studies in order to assess the overall evidence of space–time clustering for different childhood cancers. This review includes studies of incidence or mortality for pre-defined study areas and thus excludes ex post investigations of putative cancer clusters. We assessed the evidence of clustering separately for individual diagnostic groups for place and time of birth and diagnosis overall as well as for age subgroups. Since many original studies reported results of Knox tests, we used a novel method for pooling test statistics from individual studies allowing us to evaluate the overall evidence and determine the spatial and temporal scales for which the strength of clustering was most pronounced. Furthermore, we defined criteria to evaluate the quality of studies in order to account for and minimize the risk of bias.

## Methodology

A detailed description of the methodology is provided in the online supplementary material.

We followed the PRISMA guidelines [15] for the reporting of this systematic review.

### Literature search

We conducted a systematic search of the Embase and MEDLINE data bases in September 2014. We subsequently received updates from this electronic search and evaluated all studies published up to June 2016. Our search included key terms pertaining to [1] neoplasms and malignancies, [2] childhood and adolescence and [3] Geographic Information Systems and spatial or space–time clustering. Additionally, we screened the bibliographies of studies included in the data extraction and of previous reviews [8–13] for eligible studies.

### Study selection

Three reviewers (CK, ED and JEL) screened titles and abstracts and subsequently scanned the full text of eligible studies. We included studies that [1] assessed clustering in space–time of specified childhood cancers by means of a statistical test, [2] included children and adolescents < 20 years of age and [3] identified cases from a population-based cancer registry or a data base with reliable case ascertainment for a pre-defined study area. In the pooled analysis, we included studies that reported details of a Knox test including the number of observed and expected close pairs of cases.

### Data extraction

Two reviewers (CK and ED) independently extracted the data on the study population, area, period and design, diagnostic groups, clustering tests performed and results from each study (the list of extracted items is provided in the supplementary material). For studies included in the pooled analysis, we retrieved the data from each individual Knox test reported including the spatial and temporal lags and the observed and expected number of close pairs. We used EpiData (Version 3.1.) to extract the data and to compare extractions and reach consensus.

### Statistical methods

In order to gauge the strength of the evidence, we calculated the proportion of significant space–time clustering tests for each diagnostic group. We thus recorded for each original study the number of significant tests (*p* < 0.05) and divided this number by the total number of tests performed. If a given study performed one single test (e.g. K-functions) or adjusted for multiple testing, this proportion was equal to 1 if there was evidence of clustering and 0 otherwise. We then calculated the mean proportion over all included studies and refer to this value as the mean proportion of significant tests at the 5% alpha-level (MPST). In the absence of space–time clustering and in the absence of biases, the expected values of MPST is 5%, with higher values supporting the presence of clustering, although the reliability of this measure depends on it being based on a large number of studies. Studies using scan statistics as statistical test which are meant to detect localized excesses of cases as opposed to a general tendency of cases to occur more closely in space and time than expected by chance were excluded from this evidence synthesis (see Methodology section in the online supplementary material).

For the pooled analysis, we exploited the fact that the test statistic of the Knox test is approximately Poisson distributed [16] and that the sum of independent Poisson deviates also follows a Poisson distribution. Let $O_{i}$ be the observed number of pairs of cases in study $i$ that lie close to each other in space and time (for specified spatial and temporal lags), i.e. the Knox statistic, and $E_{i}$ its expected value in the absence of space–time clustering. Then the summary test statistic $\sum\nolimits_{i = 1}^{n} {O_{i} }$ for $n$ independent studies is approximately Poisson distributed with mean $\sum\nolimits_{i = 1}^{n} {E_{i} }$. In addition, we computed $S_{i} = 100\left( {O_{i} - E_{i} } \right)/E_{i}$, i.e. the relative excess number of close pairs in percent, as a measure of the strength of clustering [7].

We performed analyses separately for individual diagnostic groups for place and time of birth and diagnosis. For childhood leukaemia and ALL, we additionally performed separate analyses for age subgroups (0–5, 5–15 years) as well as pooled analyses for predefined spatial and temporal scales. We also performed separate analyses by study period, study region, size of study, clustering test and by four quality criteria which, if not met, reduce power or risk bias in space–time clustering analyses: high coverage of case ascertainment, high spatial resolution of address geocoding, correction for multiple testing and adjustment for uneven shifts in the background population. For each analysis, we selected one test per eligible original study, excluding, to the extent possible, studies with overlapping data. For some earlier studies, which used mortality data, time of death was treated as time of diagnosis. All analyses were performed using the R language for statistical computing (Version 3.4.0).

A detailed description of the methodology is provided in the online supplementary material.

## Results

**Fig. 1:**
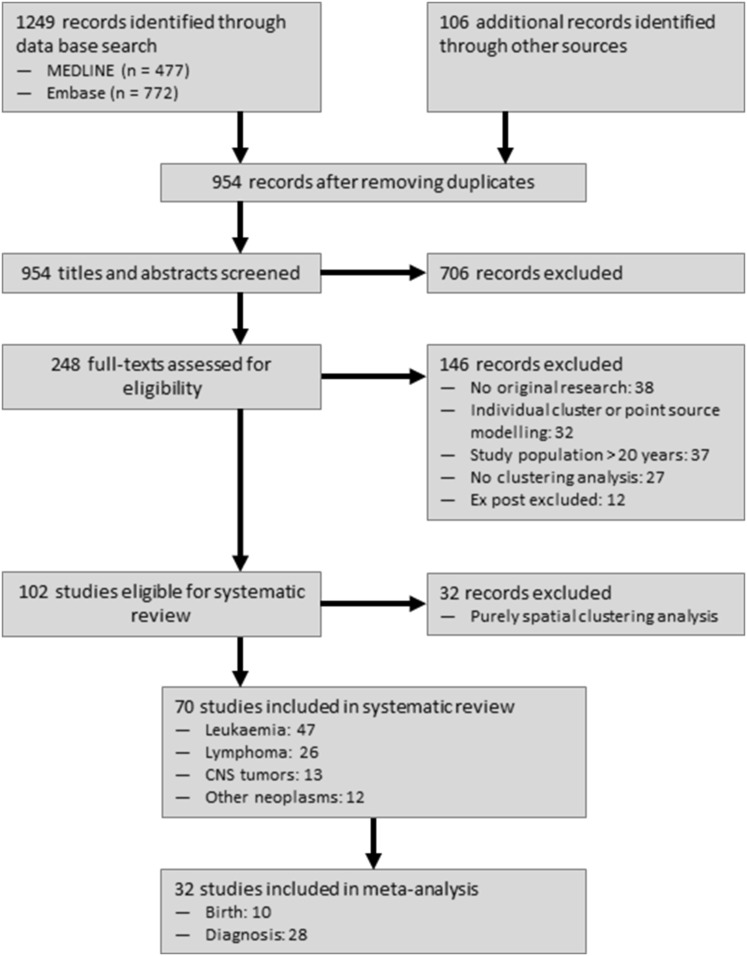
Flow diagram of study selection

Reviewing 954 titles and abstracts and scanning 248 full texts, we identified 70 space–time clustering studies that met our inclusion criteria. Thereof, 32 studies reported Knox tests of childhood cancers and were included in our pooled analysis (Fig. 1). The year of publication ranged from 1959 to 2016, and the overall sample sizes varied between 29 and 32,323 cases of childhood cancer.

### Leukaemia

The characteristics of the 47 identified studies of childhood leukaemia are presented in Table S1 in the online supplement. 18 studies were conducted in the USA, 15 in the UK, 11 in continental Europe, 2 in New Zealand, 1 in Pakistan and 1 was an international study [17]. The pioneering US and UK studies with two exceptions [18, 19] included fewer than five hundred cases, yet since the 1990s several state- or nationwide studies of more than one thousand cases have been carried out in the USA, the UK and continental Europe.

The results of these space–time clustering studies are summarized in Table 1. A total of 41 studies analysed clustering of childhood leukaemia for place and time of diagnosis, 13 of which [20–32] were excluded due to overlapping data samples. A further 5 studies [33–37] employing space–time scan statistics were dropped from the evidence synthesis. Of the remaining 23 studies [6, 7, 18, 19, 38–56], 10 individually were suggestive of clustering, i.e. their proportions of significant tests to the total number of tests performed exceeded 0.05 or 5%. Over these 23 studies the mean proportion of significant tests (MPST) was 26%. For place and time of birth, after excluding 2 studies [21, 22] due to overlapping data, only 1 out of 9 studies [7, 40, 41, 47, 56–60] reported evidence of clustering (MPST = 11%). Stratified by age subgroup, among children under 5 years of age there was support of clustering at diagnosis (MPST = 26%) but not at birth (MPST = 0%), whereas for children aged 5–15 years there was little support of clustering both at birth (MPST = 0%) and diagnosis (MPST = 0%, Table 1).

**Table 1:** Quantitative synthesis of results of space–time clustering analyses of childhood cancers for time of diagnosis and birth: number of studies individually reporting significant evidence of space–time clustering and mean proportion of significant tests across studies (MPST)

Diagnostic group	Diagnosis		Birth	
	n^a^/No^b^	MPST^c^ (%)	n^a^/No^b^	MPST^c^ (%)
Leukaemia	10/23	26	1/9	11
Leuk 0–15	10/23	26	1/5	20
Leuk 0–5	5/12	26	0/8	0
Leuk 5–15	0/5	0	0/3	0
ALL	4/11	23	1/7	1
ALL 0–15	4/10	25	0/5	0
ALL 0–5	1/7	14	1/6	1
ALL 5–15	0/4	0	1/3	7
AML	1/6	1	0/2	0
Lymphoma*	2/7	8	0/3	0
HL	1/4	13	1/2	50
NHL	2/4	14	0/2	0
Burkitt lymphoma	3/8	13	0/0	
CNS	1/7	7	1/3	33
Astrocytoma	1/4	19	0/2	0
Ependymoma	0/2	0	0/1	0
PNET	1/2	29	0/2	0
Other cancers				
Neuroblastoma	1/3	17	0/2	0
Retinoblastoma	0/2	0	0/1	0
STS	1/2	50	0/2	0
Renal	0/2	0	1/2	38
Bone tumors	1/3	4	0/2	0
Osteosarcomas	2/3	37	1/2	50

Diagnostic group: *ALL* acute lymphoblastic leukaemia, *AML* acute myeloid leukemia, *HL* Hodgkin’s lymphoma, *NHL* non-Hodgkin’s lymphoma, *PNET* primitive neuroectodermal tumors, *STS* soft tissue sarcoma

*Excluding studies focusing exclusively on Burkitt lymphoma

^a^Number of studies individually reporting significant evidence of clustering (proportion of significant clustering tests > 0.05)

^b^Number of studies included (excluding studies with overlapping samples and studies employing scan statistics as clustering test)

^c^Mean proportion of significant clustering tests across included studies

Results of clustering analyses of ALL were broadly similar. There was support of space–time clustering at diagnosis overall [6, 7, 40–42, 46, 47, 49, 51, 55, 56] (MPST = 23%) but only 1 out of 7 studies indicated clustering for children aged 0–5 years (MPST = 14%) and none for older children. At birth, there was little evidence of clustering both overall and stratified by age though [7, 40, 41, 47, 56–58]. By contrast, 7 studies assessing cases of AML [6, 7, 20, 46, 49, 55, 57] reported virtually no evidence of clustering neither for place and time of birth nor diagnosis (Table 1).

### Lymphoma

Of 26 studies of childhood lymphoma, 9 studies were carried out in the UK, 9 in Africa, 5 in the USA and 3 in continental Europe (Table S2). The earliest studies primarily analysed Burkitt lymphoma in Uganda [61, 62], Tanzania [63] and Ghana [64], whereas contemporaneous studies from the USA and UK were small and did not always analyse lymphoma as a distinct diagnostic group but lumped them together with leukaemia. Since 1990 there have been several state- or nationwide clustering analyses in the USA, the UK and continental Europe, some of which still combined cases of leukaemia and lymphoma though.

Results of studies of lymphoma (excluding those focusing exclusively on Burkitt lymphoma [61–69], those combining lymphoma and leukaemias [22, 23, 41], and dropping 3 studies because of overlapping data [20, 70, 71] and 3 scan statistics studies [34, 36, 37]) provided only weak support of space–time clustering at time of diagnosis (Table 1). Little support for clustering came from the complete analysis [32, 45, 49, 51, 56, 72, 73] (MPST = 8%) but there was some support of clustering in separate analyses of both Hodgkin’s lymphoma [45, 51, 72, 73] (MPST = 13%) and non-Hodgkin’s lymphoma [45, 49, 51, 72] (MPST = 14%), albeit based on only 1 and 2 positive studies, respectively. At time of birth, none of three studies [56, 57, 72] analysing all lymphomas combined reported evidence of clustering. There was some evidence of clustering of Hodgkin’s lymphoma (MPST = 50%) but this was based on only 2 studies with partly overlapping data [57, 72]. For Burkitt lymphoma, there was some indication of space–time clustering at time of diagnosis based on 3 positive out of a total of 8 studies (MPST = 13%, Table 1). All 8 of these studies [61, 63–69] were conducted in African countries during 1967–1993 (Table S2).

### CNS tumours

A total of 13 space–time clustering studies of childhood brain or CNS tumours as a separate diagnostic group have been conducted since the early 1990s; 5 in the UK, 5 in continental Europe and 3 in the USA (Table S3). Excluding 1 study because of overlapping data [20] and 4 studies employing scan statistics [34, 36, 37, 74] from the evidence synthesis, results based on 7 studies [45, 51, 56, 75–78] provided little support of clustering at time of diagnosis for CNS tumours overall (MPST = 7%, Table 1). There was some support of space–time clustering of astrocytomas [51, 75, 77, 78] (MPST = 19%) and primitive neuroectodermal tumours [20, 75] (PNET) (MPST = 29%) albeit both based on only 1 positive study, whereas 2 studies investigating ependymoma [75, 77] found no evidence of space–time clustering. Only 3 studies investigated clustering at birth [56, 57, 75]. One of these studies, from the UK, was positive for CNS tumours (MPST = 33%, Table 1), but no evidence of clustering was reported for any diagnostic subgroup.

### Other cancers

A total of 12 studies separately assessed space–time clustering of other childhood cancers [20, 37, 45, 51, 56, 57, 74, 79–83]; 5 in the USA, 4 in the UK and 3 in continental Europe (Table S4). For these cancers, at most four individual studies have assessed clustering for any given diagnostic group. Excluding two studies using scan statistics [37, 74], some support for clustering was found at diagnosis for osteosarcoma [51, 79, 81] (MPST = 37%), neuroblastoma [56, 79, 82] (MPST = 17%) and soft tissue sarcomas [51, 56] (MPST = 50%) and at time of birth for osteosarcoma [57, 79] (MPST = 50%) and renal tumours [51, 79] (MPST = 38%). By contrast, none of the two studies of retinoblastomas [79, 80] found evidence of clustering (Table 1).

### Pooled analysis of studies using Knox tests

**Fig. 2:**
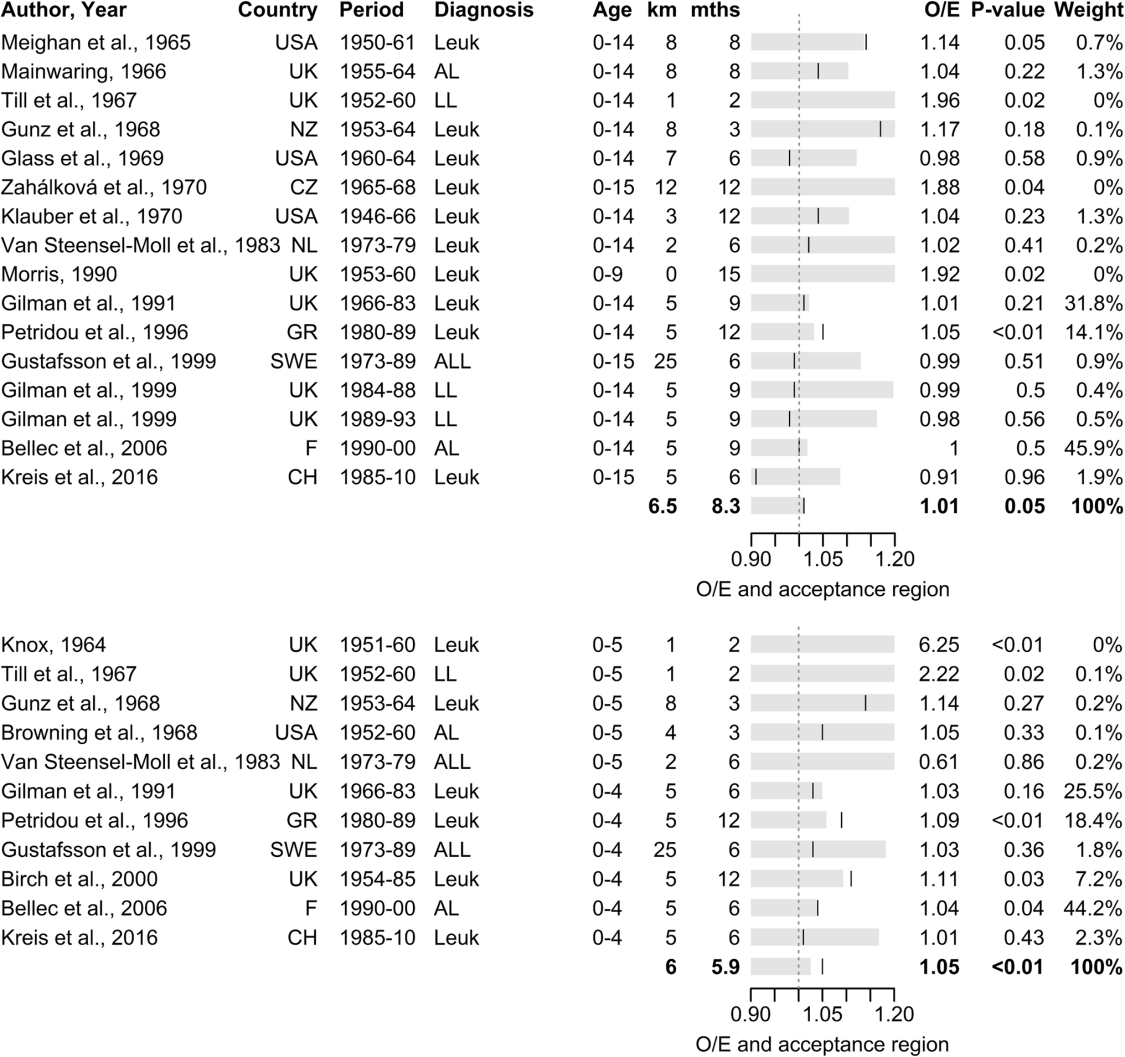
Pooled analysis of space–time clustering studies of childhood leukaemia for place and time of diagnosis for children aged 0–15 years (top) and 0–5 years (bottom): forrest plot of ratio of observed over expected number of close pairs of cases (O/E) and acceptance region for one-sided Knox test at 5% alpha-level assuming Poisson distribution

For childhood leukaemia at time of diagnosis, a pooled analysis of 16 individual samples from 15 original studies [21, 24, 25, 27, 38, 42, 44, 46, 47, 49, 50, 53, 54, 56, 84] showed marginally significant space–time clustering (*p* = 0.054) with an overall excess of close pairs of S = 1.1% (Table 2, Fig. 2). This result was largely driven by 2 large studies from France [38] and the UK [49] showing no evidence of clustering, and a Greek study [42] that found strong evidence of clustering (Fig. 2). For time of birth, a pooled analysis of 6 individual samples from 5 studies [7, 22, 40, 41, 56] with non-overlapping data showed little evidence of space–time clustering. The summary *p* value was 0.395, corresponding to an overall excess of close pairs of 0.3% (Table 2, Figure S2).

**Table 2:** Summary table of results of the pooled analyses of space–time clustering studies of childhood cancers by diagnostic group and age subgroups

Diagnostic group	Diagnosis					Birth				
	km/mths^a^	Studies	Excess^b^ (%)	Obs^c^	*p* value^d^	km/mths^a^	Studies	Excess^b^ (%)	Obs^c^	*p* value^d^
Leukaemia	6.5/8.3	16	1.1	20,107	0.054	10/12	6	0.3	7870	0.395
0–5 years	6/5.9	11	5.2	4603	< 0.001	9.6/9.6	5	4.6	1169	0.06
5–15 years	15/12	3	− 1.9	6703	0.938	17.5/12	2	3.2	316	0.278
ALL	6.6/7.4	8	0.4	6369	0.359	11.2/12	4	0.6	2415	0.382
0–5 years	7.1/9.4	6	5.3	1235	0.035	9.6/9.6	5	3.9	956	0.116
5–15 years	17.5/12	2	− 13.2	241	0.986	17.5/12	2	3.5	225	0.289
Lymphoma	5/12	4	2.8	582	0.243	5/12	2	− 1.8	157	0.57
CNS	5/12	4	3.2	1431	0.114					
Neuroblastoma						5/12	2	2.7	77	0.38

^a^Mean spatial and temporal lags of Knox tests pooled across studies

^b^Excess number of close pairs of cases observed across studies in excess over the number expected under the assumption of no space–time clustering expressed as a percentage of the number expected: S = 100 * (O − E)/E

^c^Number of close pairs of cases observed across studies

^d^One-sided Knox test of the number of close pairs of cases observed against the number expected across pooled studies assuming Poisson distribution

For leukaemia at age 0–5 years, strong evidence of clustering for time of diagnosis was found based on a pooled analysis of 11 studies [6, 7, 21, 27, 38, 42, 46, 47, 49, 52, 56]. All but one study found more close pairs than expected, producing an overall excess of close pairs of 5.2% (*p* < 0.001, Table 2, Fig. 2). For time of birth, a pooled analysis of 5 studies [7, 41, 47, 56, 58] found marginally significant clustering, with each study individually showing an excess of close pairs accounting for an overall excess of 4.6% (*p* = 0.06, Table 2, Figure S2). For children aged 5–15 years, by contrast, a pooled analysis of 3 studies for time of diagnosis [21, 49, 56] revealed a shortage of close pairs of cases of 1.9% below that expected (*p* = 0.938, Table 2, Figure S1). For time of birth, a pooled analysis of just 2 studies [41, 56] produced no evidence of clustering (*p* = 0.28, S = 3.2%, Table 2, Figure S2).

**Fig. 3:**
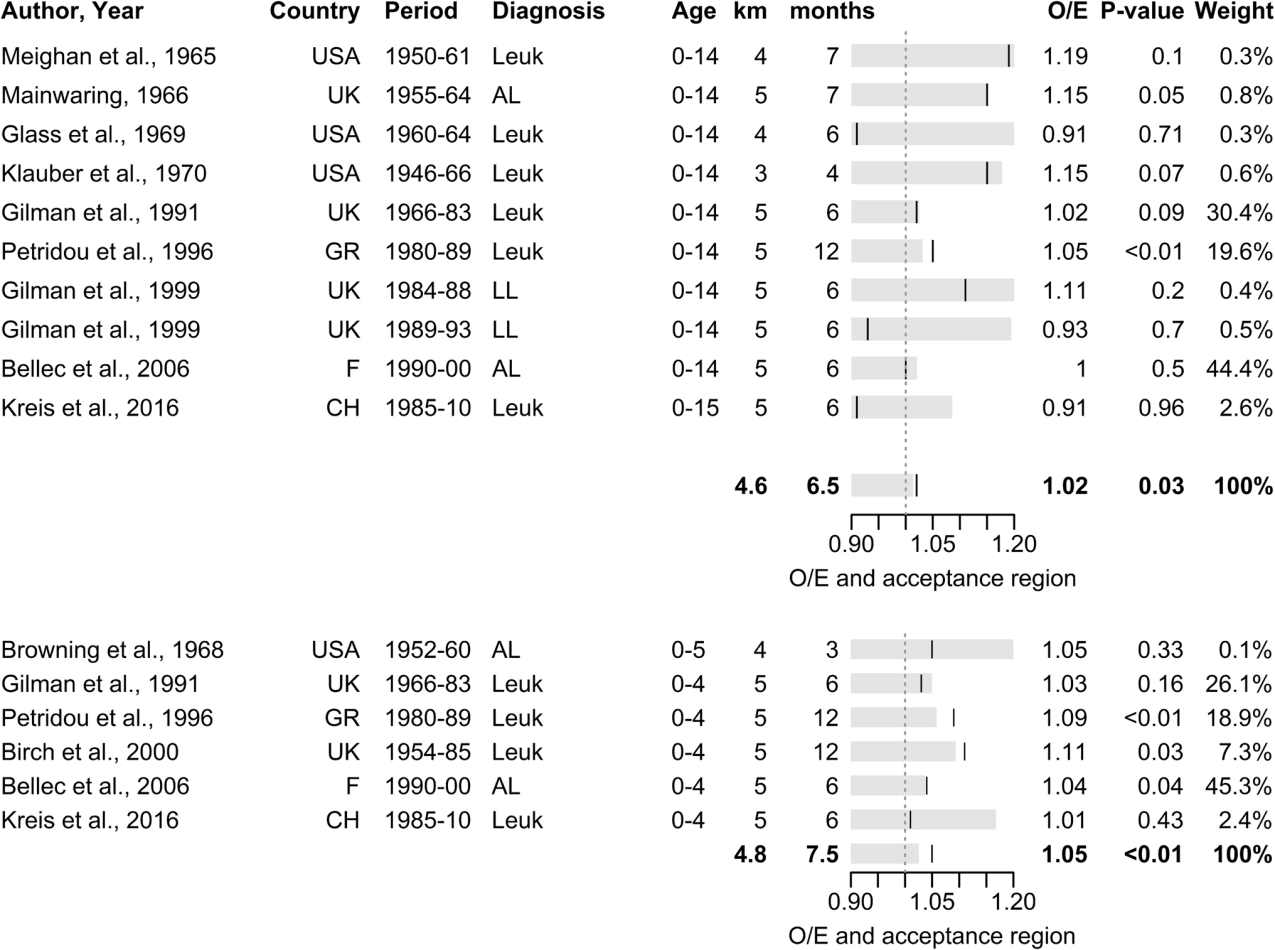
Pooled analysis of space–time clustering studies of childhood leukaemia for place and time of diagnosis for children aged 0–15 years (top) and 0–5 years (bottom) for spatial lags of 3–7 km and temporal lags of 0–12 months: forrest plot of ratio of observed over expected number of close pairs of cases (O/E) and acceptance region for one-sided Knox test at 5% alpha-level assuming Poisson distribution

When investigating for clustering at different spatial and temporal scales, strongest evidence was found in 0–5 year olds at diagnosis using temporal lags of 3–7 km and spatial lags of 0–12 months (S = 5.2%, *p* < 0.001, Table 3, Fig. 3). Weaker evidence was found for longer spatial or temporal lags (Table 3, Figure S3–S5) or for the same lags when extending to the full age range 0–15 years (S = 1.6%, *p* = 0.026, Table 3, Fig. 3). For time of birth, pooled analyses showed no evidence of clustering for any given set of spatial and temporal lags. However, for children aged 0–5 years, an extraordinary but non-significant excess of 10–14% was found for spatial lags of 0–3 km (Table 3).

**Table 3:** Summary table of results of the pooled analyses of space–time clustering studies of childhood leukaemia for age groups 0–15 and 0–5 years by different ranges of the spatial and temporal lags of the pooled Knox tests

Age group	Diagnosis					Birth				
	km/mths^a^	Studies	Excess^b^ (%)	Obs^c^	*p* value^d^	km/mths^a^	Studies	Excess^b^ (%)	Obs^c^	*p* value^d^
0–15 years	0–3/0–12	12	0.8	4707	0.299	0–3/0–12	3	− 0.6	608	0.548
	0–3/6–18	11	0.3	8796	0.378	0–3/6–18	3	0.2	1158	0.471
	0–3/12–36	10	0.9	10,739	0.164	0–3/12–36	3	2.9	1317	0.151
	3–7/0–12	10	1.6	14,480	0.026	3–7/0–12	5	− 1.1	4075	0.752
	3–7/6–18	10	1.7	14,035	0.025	3–7/6–18	5	− 0.8	6487	0.743
	3–7/12–36	10	1.4	22,892	0.015	3–7/12–36	5	− 0.5	6963	0.674
	7–15/0–12	8	0.4	32,718	0.257					
	7–15/6–18	7	0.4	61,955	0.184					
	7–15/12–36	7	0.6	88,314	0.033					
0–5 years	0–3/0–12	8	1.8	1473	0.238	0–3/0–12	3	14.2	612	0.128
	0–3/6–18	4	0.5	2734	0.398	0–3/6–18	3	9.9	621	0.126
	0–3/12–36	3	0.5	3196	0.377					
	3–7/0–12	6	5.2	4495	<0.001	3–7/0–12	4	3.7	8796	0.194
	3–7/6–18	5	2.7	7268	0.011	3–7/6–18	4	0.9	10,739	0.406
	3–7/12–36	5	1.9	9353	0.035	3–7/12–36	2	− 1.5	14,480	0.641
	7–15/0–12	4	2.0	9026	0.027					
	7–15/6–18	3	0.4	16,866	0.313					
	7–15/12–36	3	0.5	23,424	0.218					

^a^Range of spatial and temporal lags of Knox tests pooled across studies

^b^Excess number of close pairs of cases observed across studies in excess over the number expected under the assumption of no space–time clustering expressed as a percentage of the number expected: S = 100 * (O − E)/E

^c^Number of close pairs of cases observed across studies

^d^One-sided Knox test of the number of close pairs of cases observed against the number expected across pooled studies assuming Poisson distribution

Results were similar for ALL though pooled analyses were based on fewer studies [7, 21, 25, 40–42, 46, 47, 49, 56, 58]. Again, strongest evidence of clustering was found at time of diagnosis for children aged 0–5 years (S = 5.3%, *p* = 0.035, Table 2, Figure S6). For older children aged 5–15 years, a pooled analysis of 2 studies [21, 56] revealed a substantial shortage of close pairs of cases (S = − 13.2%, Table 2, Figure S6). Pooled analyses revealed no evidence of clustering around time of birth (Table 2, Figure S7).

Pooled analyses of studies of lymphoma [49, 56, 70, 72], CNS tumours and neuroblastoma [56, 79] showed no evidence of space–time clustering (Table 2, Figure S8). However, these included fewer studies. For CNS tumours, a pooled analysis of 4 individual samples from 3 studies [56, 76, 77] found an overall excess of cases of 3.2% for time of diagnosis (*p* = 0.114).

### Risk of bias and sensitivity analysis

Quality assessments of included studies are presented in the characteristics tables in the online supplementary material (Table S5). Since the 1980s most studies obtained incidence data from specialized regional or national cancer registries, diminishing the risk of bias due to incomplete case ascertainment and low temporal resolution. The use of exact geocodes on the other hand remains rare with most studies relying on postcodes or small administrative areas as georeference. Moreover, only few studies corrected for multiple tests performed for different combinations of spatial and temporal lags for different diagnostic groups. Since the late 1990s this problem has been attenuated though, as most studies either accounted for multiple testing directly or, more commonly, used methods such as K-functions [85] or scan statistics [86] that perform only a single test. Also, only few studies attempted to adjust for uneven shifts in the background population.

Sensitivity analyses to assess related risk of bias for childhood leukaemia are presented in the online supplementary material (Table S6). Support for space–time clustering at time of diagnosis remained strong when stratifying by region, sample size and when restricting the analyses to high quality studies. In analyses including only studies with a methods score of 3 or higher or those correcting for shifts in the background population, the evidence of clustering was comparable or even stronger than in the analyses including all studies. We also conducted two separate analyses of only the most recent studies, one in which we included studies whose study period began in 1980 or later and another of studies whose study period ended in 1990 or later. In both analyses, support for space–time clustering was reduced but MPST was still far above the level expected by chance in the absence of any space–time interaction. We further ran separate analyses by clustering test, including one of Kulldorff’s scan statistics cluster detection test (Table S6). Finally, we found little evidence of selective reporting of the results of Knox tests. Most studies reported the number of observed and expected close pairs for every combination of space–time lags examined (results not shown).

## Discussion

This first systematic review and pooled analysis of space–time clustering of childhood cancers provides new evidence that cases of leukaemia cluster in space and time. The strongest evidence was found for children aged 0–5 years based on address of residence at diagnosis: The mean proportion of significant tests across 12 studies was 26% and the pooled Knox test revealed a relative excess of pairs of cases that were close to each other in time and space of more than 5%. For the age group 5–15 years both MPST and the pooled analysis using residence at diagnosis were suggestive of a deficit rather than an excess of close pairs. Little evidence of space–time clustering at the time of birth was found. Results from studies of cases of ALL were broadly similar.

By contrast, the evidence in support of space–time clustering for other diagnostic groups was mixed. For lymphoma (excluding studies focusing exclusively on Burkitt lymphoma) and CNS tumours there was little evidence of clustering overall based on the quantitative synthesis of the systematic review and the pooled analyses of studies using Knox tests. For some diagnostic subgroups such as Hodgkin lymphoma, non-Hodgkin lymphoma, astrocytoma, and PNET there was an excess of significant tests above expectation, however based on only one or two positive studies and we had insufficient data to perform pooled analyses. For other solid cancers, there was some indication of clustering of osteosarcomas whereas for some diagnostic groups such as retinoblastomas no previous study produced evidence of space–time clustering.

Our results are broadly consistent with the findings of previous narrative reviews regarding childhood leukaemia which found strong evidence of clustering at diagnosis and were also suggestive of clustering at birth [11–13]. By contrast, our results differ with regard to other diagnostic subgroups of lymphoma, CNS tumours and other rarer solid tumours for which, based on a broader evidence base, we do find some indication of space–time clustering.

Our study is the first systematic review and pooled analysis of space–time clustering studies of childhood cancers. Given the large number of original studies and the diversity of methodologies used, a qualitative synthesis of the evidence would be prone to subjective judgement. We therefore relied on objective criteria to gauge the evidence for different diagnostic groups and age subgroups at the time points of birth and diagnosis. Specifically, we calculated the mean proportion of significant clustering tests across studies for each diagnostic group which allowed combining the evidence from different statistical tests while simultaneously reducing the risk of finding spurious evidence due to multiple testing. This correction for multiple testing could not take into account the correlation between individual clustering tests, though, and might therefore have been overly conservative. For instance, Knox tests are typically performed for multiple spatial and temporal lags such that the close pairs for smaller lags are a subset of those for bigger lags resulting in correlated tests. If these tests are perfectly correlated, they essentially contain the same information as a single test, i.e. a study with 10 significant tests out of 10 tests provides equally strong evidence as a study with 1 out of 1 significant test. However, if tests are only weakly correlated, each of the 10 tests of the first study contributes to the overall evidence which will be stronger than that of the study with only 1 test. Because MPST only takes into account the proportion of significant tests, both studies contribute equally even if 10 out of 10 significant tests represents stronger evidence when tests are only weakly correlated. Furthermore, we propose a meta-test for evidence synthesis of studies using the Knox test. This method allows calculating the overall excess of close pairs across studies as a crude measure of clustering strength, enabling us to compare this measure across different spatial and temporal lags in order to assess the spatial and temporal scales of clustering. Finally, we also assessed the quality of included studies using a set of criteria, which, if not met, risk producing spurious evidence of clustering.

Inevitably, our study has some limitations. Because of the large differences between studies in the number and nature of tests performed, evidence synthesis for any particular combination of diagnostic group, age group and time frame was often based on a small selection of studies particularly for time of birth. Differences in the strength of evidence between different combinations might thus in part be due to the different sets of included studies. Further, research from the UK had an outsized influence in terms of both the number of original studies and the number of childhood cancer cases included, and the partly overlapping study samples made it at times unavoidable either to lose or double-count cases. In the pooled analysis we could only include studies reporting the observed and expected number of close pairs of individual Knox tests, and thus had to exclude a number of recent studies using K-functions. Moreover, our evidence synthesis could not take into account the possibility of heterogeneity between study populations. We assumed that a tendency of a cancer type to cluster in space and time would be similarly manifest in each population. However, such a tendency, particularly if it is causally related to an infectious agent, might depend on population specific attributes such as climate, population density, socio-economic status, ethnicity etc. Furthermore, any biases present in the included original studies will also have affected our evidence synthesis. However, we did not find any marked difference in studies of childhood leukaemia adjusting for some well-known sources of bias in clustering studies captured by our four quality criteria (see Methodology section in the online supplementary material). On the contrary, in analyses including only studies of good quality or those correcting for shifts in the background population, the evidence of clustering was comparable or even stronger than in the analyses including all studies. We also were unable to assess or account for the risk of publication bias because we could not calculate a standardized effect size across studies. Nevertheless, we think it is unlikely that publication bias, if present, would have altered our main conclusions as the pooled Knox tests give smaller studies proportionally less weight than traditional effect size meta-analysis. Lastly, we could not account for risk of bias in the cumulative evidence due to the evolving diagnostic procedures, coding and classification of neoplasms over the years, notably of the leukaemias and lymphomas which taken together account for a sizeable share of paediatric cancers [87]. This systematic review included several studies, the study period of which spanned two or more decades during which diagnostic and registration procedures have altered substantially and which may falsely contribute to the evidence of space–time clustering as a result. In our evidence synthesis, we aimed to be inclusive and incorporated studies regardless of the beginning or length of study period or of changes to disease classification that became effective during this time. In order to assess the ensuing risk of bias, we performed sensitivity analyses including only the most recent leukaemia studies. We found the proportion of significant clustering tests was somewhat reduced compared to the analyses of all studies but remained far above the level expected from a chance distribution of cases, suggesting that the risk of bias from these changes in classification and registration is unlikely to change the overall conclusions.

The most robust evidence of space–time clustering was found for leukaemia at time of diagnosis among children under 5 years of age. This finding was strongly supported by a pooled analysis of 11 studies covering eight countries across three continents and six decades, the four largest of which all suggested excesses of close pairs of cases in the range of 3–11%. This suggests that the clustering may indeed be due to some ubiquitous aetiological factor that primarily affects children under the age of 5 years, an age window that includes the age of peak incidence at 2–5 years. The critical spatial and temporal lags of the Knox tests maximizing the strength of clustering were 5 km and 6 months, respectively. Notwithstanding the inherent limitations when pooling data across continents, this suggests that the putative aetiological factor causing the clustering has a short range of few kilometres and is short-lived. This small spatial and short temporal scale of the clustering would be compatible with exposure to common infections that come about as short and localised mini-epidemics as the aetiological factor.

Assuming a short latent period between exposure and onset of disease, the observed clustering pattern fits well with Greaves’ delayed-infections hypothesis. Greaves, seeking to explain the characteristic childhood peak incidence of ALL, posited a ‘two-hits’ model in which a first hit occurring in utero induces chromosomal changes in a precursor B-cell that is passed on to daughter cells. A lack of exposure to infections in early infancy is hypothesized to result in an aberrant response to ‘delayed’ infections (the second hit) prompting the onset of overt disease [88]. Following a lack of exposure in the first year of life, children could be particularly susceptible to the second hit around the age of 2–4 years. A high circulation rate of the putative infection in a given area could result in an initial excess of cases in this cohort of children, followed by a shortage as the cohort grows older due to the early depletion of susceptibles. This would for instance explain why the strongest clustering signal is observed for the age 0–5 years at diagnosis: this age window includes the period of prime susceptibility to the putative aetiological factor and the period of peak incidence, which translates into increased statistical power of space–time clustering tests. For the ages 0–15 years clustering is still positive but weaker, possibly because it is diluted through partial depletion of susceptibles and residential mobility. The said hypothesis might also offer a plausible explanation of the shortfall of close pairs observed at the age of 5–15 years, which was most noticeable for ALL.

This clustering scenario could also account for the space–time clustering around time of birth observed in a couple of studies. If the putative aetiological factor operates closer to the time of diagnosis but at a young age, we would expect to observe some clustering also at birth provided that only a small proportion of children will have moved residence between the two time points. However, if the time window of susceptibility to this aetiological factor extends over several years (e.g. 2–4 years as assumed above), the strength of clustering might be diluted compared to time of diagnosis and be more likely to show up in studies with larger samples or that use long time-lags in the clustering test. In other words, the null finding of many studies for time of birth could be the result of the small study samples and short temporal lags used in the clustering tests.

Overall, the evidence from this systematic review thus makes exposure to infections during the childhood peak years appear likely as the aetiological factor driving the space–time clustering. However, based on the evidence we cannot rule out that exposure to environmental pollution from local sources with time-varying emission levels (partly) account for the observed clustering. Also, the strength of the observed clustering is small—generating few extra close cases above expectation in a random distribution—but rather persistent across study areas and periods. It is possible that the aetiological factor driving the clustering may only affect some leukaemia subtypes. Under this scenario, analyses combining all types may dilute the evidence of clustering. This interpretation is compatible with previous findings which observed strong clustering for the precursor B-cell subtype of ALL [40], notably of the cytogenetic subtype of ETV6-RUNX1 fusion [89] even though this latter finding still awaits replication.

The evidence of space time clustering for other diagnostic groups was mixed. However, the absence of evidence of space–time clustering cannot be interpreted as evidence of the absence of any space–time interaction. First, evidence of clustering is more likely to be detected if the latency period following the critical exposure is short or relatively constant. This might render it more difficult to detect any clustering for lymphomas, CNS tumours and other solid tumours for which latency is longer than for leukaemia and also the delay between first symptoms and diagnosis may be longer and vary greatly between cases. Secondly, evidence synthesis for this systematic review was based solely on studies assessing global space–time clustering, i.e. a general tendency of cases to occur more closely in space and time than expected under the assumption of independence of spatial and temporal incidence patterns. In a complementary analysis of studies trying to detect space–time clusters using Kulldorff’s scan statistics, we did observe a noticeably higher proportion of significant tests particularly for CNS tumours and, to a lesser extent, lymphomas (Tables S3–S4). This would suggest that the little evidence of space–time clustering of these diagnostic groups does not indicate the absence of any space–time interaction per se but could be the result of an incidence pattern distinct from leukaemia that involves few localized space–time clusters which are more likely picked up by cluster detection tests as compared to a global test of clustering. Furthermore, fewer clustering studies have been conducted for childhood cancers other than leukaemia and sample sizes were often smaller. For many of these diagnostic groups the evidence base, particularly for the time point of birth, is still too small to draw firm conclusions.

In conclusion, combining the evidence from five decades of clustering studies, this systematic review and pooled analysis found clear evidence of space–time clustering of childhood leukaemia. It is particularly evident during the age of peak incidence around time of diagnosis, suggesting an early age window of heightened susceptibility to the aetiological factor and a short latency. The spatial and temporal scales maximizing the strength of clustering imply that the aetiological factor is short-lived and short-ranged. The observed pattern is compatible with Greaves’ delayed-infections hypothesis. The evidence base for other diagnostic groups remains narrow, allowing no firm conclusions.

## Electronic supplementary material

Below is the link to the electronic supplementary material.

Supplementary material 1 (DOCX 102 kb)

Supplementary material 2 (PDF 1657 kb)

Supplementary material 3 (PDF 137 kb)

Supplementary material 4 (PDF 135 kb)

Supplementary material 5 (PDF 117 kb)

Supplementary material 6 (PDF 127 kb)

Supplementary material 7 (PDF 90 kb)

Supplementary material 8 (PDF 85 kb)
