# Supplementary material for: Space–time clustering of childhood cancers: a systematic review and pooled analysis
Source: Eur J Epidemiol. 2018 Nov 16;34(1):9–21. doi: 10.1007/s10654-018-0456-y (PMC6326085; doi:10.1007/s10654-018-0456-y)
Supplement: Supplementary file 1 — Supplementary material 1 (DOCX 102 kb) [file 10654_2018_456_MOESM1_ESM.docx]

**Supplementary material**

**Space-time clustering of childhood cancers: a systematic review and pooled analysis**

**Christian Kreis^1^, Eliane Doessegger^1^, Judith E. Lupatsch^1^ and Ben D. Spycher^1^**

1. Institute of Social and Preventive Medicine (ISPM), University of Bern, Bern, Switzerland

**Corresponding author:**

Ben D. Spycher

Postal address: University of Bern, Institute of Social and Preventive Medicine (ISPM), Mittelstrasse 43, 3012 Bern, Switzerland

Tel. +41 31 631 33 46

Fax +41 31 631 35 20

Email: [ben.spycher@ispm.unibe.ch](mailto:ben.spycher@ispm.unibe.ch)

# Methodology

We followed the PRISMA guidelines (Preferred Reporting Items for Systematic Reviews and Meta-Analyses) [[2](#_ENREF_2)] for the reporting of this systematic review. We did not register and make publicly available the review protocol used for this systematic review.

## Literature search

We conducted an electronic search of the MEDLINE and Embase data bases using the Ovid search platform on September 2, 2014. We subsequently received monthly alerts from the saved search strategy and evaluated all studies published up to June 2016. Our search strings combined indexing terms (MeSH terms and keywords) on (1) neoplasms and malignancies, (2) childhood and adolescence and (3) Geographic Information Systems (GIS) and spatial or spatio-temporal clustering (the exact search strings are provided in Appendix 1). In addition, we screened the bibliographies of all studies included in the data extraction and of previous reviews [[3-8](#_ENREF_3)] for eligible studies.

## Study selection

Three reviewers (CK, ED and JEL) screened titles and abstracts of all citations identified by the electronic search according to the four-eyes-principle. The same reviewers subsequently scanned the full text of all potentially eligible studies. If two reviewers disagreed on an abstract, the full text was scanned; in case of disagreement over a full text, the two reviewers discussed it between themselves or with the principal investigator (BDS) to reach a final decision. Citations identified from the bibliographies of included studies were subjected to the same screening and selection process.

We included studies that met the following criteria: (1) the study assessed clustering in space-time of specified childhood cancers by means of a statistical test; (2) the study population included children and adolescents <20 years of age, or the study at least reported separate analyses for age subgroups <20 years; and (3) the cancer cases were identified from a population-based cancer registry or a data base with reliable ascertainment for a pre-defined study area. To be eligible for inclusion in the pooled analysis, studies had to perform a Knox test to assess space-time clustering and report the number of observed and expected close pairs directly or provide sufficient information for the latter to be inferred.

We excluded references that (1) presented no original research (e.g. abstracts, reviews, editorials, letters-to-the-editor); (2) investigated previously reported suspected cancer clusters or modelled the effect of pre-identified pollution sources; (3) examined an adult study population; or (4) did not perform an appropriate test for space-time clustering but rather investigated purely spatial or temporal clustering, seasonal variation or symptom clusters. We further excluded ex post during data extraction studies that analysed all cancers combined and did not report any results for specific diagnostic groups [[10-13](#_ENREF_10)] or assessed the cross-clustering of different childhood cancers [[14](#_ENREF_14), [15](#_ENREF_15)]; whose analyses were likely to be undermined by highly heterogeneous case ascertainment [[18](#_ENREF_18)]; focused on case aggregations among siblings [[19](#_ENREF_19)]; or did not specify the age range of the study sample [[20](#_ENREF_20), [21](#_ENREF_21)]. We additionally excluded two studies published in Hungarian [[22](#_ENREF_22)] and Chinese [[24](#_ENREF_24)] because of lacking language skills.

In the electronic search we searched for and retained studies that performed cluster detection tests, among which Kulldorff’s scan statistics is the most commonly used. Cluster detection tests seek to identify space-time clusters, i.e. localized excesses of cases in some areas at some times that cannot be explained by general excesses of cases at those locations (over the entire period) or at those times (over the entire region). By contrast, tests of space-time clustering evaluate the general tendency of cases to occur more closely in space and time than expected under the assumption of independence of spatial and temporal incidence patterns. The focus of this review is on global tests of space-time clustering and we therefore excluded cluster detection tests in our main evidence synthesis. However, we included results of Kulldorff’s scan statistics in a sensitivity analysis by clustering tests and list studies in the characteristics tables.

## Data extraction

Two reviewers (CK and ED) independently extracted the data on the study population, area, period and design, diagnostic groups, clustering analyses and results from each study. For studies included in the pooled analysis, we retrieved the data from each individual Knox test reported in a study, recording the spatial and temporal lags, the observed and expected number of close pairs, as well as the variance or standard deviation of the test statistic and p-value (if reported). The full list of extracted items is provided in Appendix 2. We used EpiData (Version 3.1.) to set up an entry mask for the data collection and to compare extractions and reach consensus [[26](#_ENREF_26)].

## Statistical methods

In order to gauge the strength of the evidence, we calculated the proportion of significant space-time clustering tests for each diagnostic group. We thus recorded for each original study the number of significant tests (p<0.05) and divided this number by the total number of tests performed. For instance, if a study performed Knox tests for four separate spatial lags and five temporal lags, and three of these Knox tests individually showed significant evidence of clustering, the proportion of significant tests was 3/(4*5)= 0.15. If a given study performed one single test (e.g. K-functions) or adjusted for multiple testing, this proportion was equal to 1 if it reported evidence of clustering and 0 otherwise. If a study used more than one method to assess clustering (e.g. Knox tests *and* K-functions or Nearest Neighbour measure *and* geographical distance), we extracted the proportion of significant tests per clustering method and took their mean as the proportion of significant tests for that study.

We then calculated the average proportion of significant tests over all included studies and refer to this value as the mean proportion of significant tests (MPST). In the absence of space-time clustering and in the absence of biases, the expected value of MPST is 5%, i.e. the conditional probability of rejecting the null hypothesis given that the null is correct. A value above the significance level of 5%, by contrast, supports the presence of clustering, although the reliability of this measure depends on it being based on a large number of studies. Calculating MPST instead of counting the number of original studies that report significant clustering tests minimizes the risk of finding spurious evidence of clustering if a number of original studies perform several clustering tests but fail to adjust for multiple testing.

We included the results of only one study sample per original study in the evidence synthesis. For instance if a study performed separate clustering tests for both leukaemia and ALL, we included only the results for the study sample of all leukaemias in the evidence synthesis for leukaemia and did not also include results for the ALL subsample in order not to double-count these cases. If on the other hand, a study analyzed *only* cases of (acute) lymphoid leukaemias, we still included the study in the analysis for all leukaemias combined. This is in accord with our specific aim here to include a maximum number of non-overlapping studies. This contrasts with our analyses of the diagnostic subgroups like ALL and AML that we wanted to keep as homogenous as possible. In these analyses we only included studies that reported specific clustering tests for these diagnostic subgroups. Likewise, if clustering tests were performed for multiple age groups, we included only one sample per study, choosing either the largest sample for the analyses comprising cases of all ages or the subsample best matching the age range of our subgroup analysis, i.e. 0-4/5 and 4/5-15 years. We deliberately chose the age brackets such as to comprise children aged 4 and 5 years at diagnosis in both subgroups in order to include a maximum of studies stratifying by age (e.g. 0-4, 0-5, 4-15, 5-15 years etc.).

## Box: Space-time clustering tests

The **Ederer-Myers-Mantel** test [[1](#_ENREF_1)] assumes that cases are randomly distributed among K cells which are appropriately defined as distinct subunits in space and time of the study area and period. The test statistic is the sum of the maximum number of cases occurring in a single cell for every time interval. The expecta­tion and variance under the null hypothesis of no space-time interaction is obtained by combinatorial ana­lysis from the multinomial distribution, with the test statistic assumed to approximate the normal distribu­tion [[9](#_ENREF_9)].

The **Knox** test [[16](#_ENREF_16), [17](#_ENREF_17)] classifies all possible pairs of cases in a study sample into a 2x2 contingency table as being close or distant in time and in space based on a pre-selected set of critical values for both geographi­cal distance (spatial lags) and the time interval (temporal lags) between cases determined by the investiga­tor. The Knox test statistic is the number of pairs of cases that are close in both space and time. This count is compared to the number of close pairs expected under the null hypothesis of no space-time interaction conditional on the marginal totals of case pairs that are close in space and close in time only. The null dis­tribution is assumed to be Poisson [[16](#_ENREF_16)] or based on the normal distribution with empirical mean and var­iance derived by Barton and David [[23](#_ENREF_23)] or a random permutation approach, whereby the times for each case are randomly reassigned while keeping locations fixed [[25](#_ENREF_25)].

**Mantel’s** test [[25](#_ENREF_25)] calculates a distance metric in both space and time between all cases in a sample and uses a reciprocal transformation thereof to derive a spatial and a temporal proximity measure which give less weight to more distant pairs of cases. The test statistic is calculated as the sum of the product of the spatial and temporal proximity measures over all possible pairs of cases in a sample. The expected value and variance of the test statistic are calculated using the random permutation approach, which for large samples approximates the normal distribution [[9](#_ENREF_9)].

**K-functions** [[27](#_ENREF_27)] calculate space-time interaction for a pre-defined set of discrete distances in space and time and may be regarded as a generalization of the Knox method. The test involves calculating the number of close pairs of cases as in the Knox test for all combinations of a set of predefined spatial and temporal lags spanning a broad range of values. The number of close pairs is then standardised by its expectation and variance under the null hypothesis of no clustering and summed over all combinations of lags to obtain the test statistic R. The null distribution of R is obtained by Monte Carlo simulation using the random permutation approach.

**Kulldorff and Hjalmars** [[28](#_ENREF_28)] showed that space-time interaction tests of case-only data are liable to be biased if population growth rates are heterogeneous across different sub-regions of the study area. They proposed a Monte Carlo method to compute unbiased tests in the face of uneven shifts of the background population.

For the pooled analysis, we pooled the test statistics from all studies that computed Knox tests for each diagnostic group, by summing the number of observed and expected close pairs, respectively, from each included study. If the spatial and temporal lags used as cut-offs for the Knox test are small, the number of expected close pairs of cases will be small compared to the total number of all possible pairs and the test statistic T approximately Poisson distributed [[16](#_ENREF_16)]. If individual studies are independent, the sum of the number of expected close pairs over all included studies will again be Poisson distributed. Formally, let $O_{i}$ be the observed number of pairs of cases in study $i$ that lie close to each other in space and time (for specified spatial and temporal lags), i.e. the Knox statistic, and $E_{i}$ its expected value in the absence of space-time clustering. In the absence of space-time clustering, the summary test statistic $\sum_{i=1}^{n} O_{i}$ for $n$ independent studies is approximately Poisson distributed with mean $\sum_{i=1}^{n} E_{i}$.

To calculate the summary test statistic, we selected one test per eligible original study. In order to ensure the independence of individual Knox tests, we excluded, to the extent possible, studies with overlapping data. In order to select comparable tests across studies, we first calculated the weighted mean spatial and temporal lags over all the Knox tests of the included studies. From each study we then took the Knox test statistic for the set of critical values closest to these mean lag values, and summed the corresponding number of observed and expected close pairs. Specifically, we calculated for every Knox test reported in a study the offset of the spatial and temporal lag from the respective mean lag in percent, and chose the set of critical values minimizing the sum of these two. Ties were broken randomly. In addition, we computed $S_{i}=100\left( O_{i}-E_{i} \right)/{E_{i}}$, i.e. the relative excess number of close pairs in percent, as a measure of the strength of clustering [[29](#_ENREF_29)].

We performed separate pooled analyses for all diagnostic groups for place and time of birth and diagnosis for which we identified a minimum of two independent studies. For childhood leukaemia and ALL, we additionally performed separate pooled analyses for different age subgroups (0-5, 5-15 years) as well as for predefined spatial and temporal scales. For these analyses, we set spatial and temporal lags of 2, 5 and 10 km and 6, 12 and 24 months, respectively, with corresponding ranges of 0-3, 3-7 and 7-15 km and 0-12, 6-18 and 12-36 months, respectively. We then selected from each original study the Knox test closest to the pre-specified lags from among all those tests whose critical values fall within the corresponding ranges. We decided to carry out these subgroup analyses *a priori*. However, we set the effective ranges based on the number of available studies. We also performed sensitivity analyses by study period, study region, size of study and the clustering test methodology including a separate test of scan statistics tests.

All analyses were performed using the R language for statistical computing (Version 3.4.0) [[30](#_ENREF_30)].

## Risk of bias

We assessed the methodological quality of included studies using four characteristics which, if not met, potentially reduce statistical power or cause bias in space-time clustering analyses: 1) high coverage of case ascertainment: we checked whether cancer cases were obtained from a population-based cancer registry or, in the absence of a registry, whether some organization maintained a central depositary of cancer cases in the study region; 2) high spatial resolution of residential address information: we recorded the spatial resolution of the geoinformation on home addresses of cancer cases used in the clustering analyses (i.e. geocodes, postcodes or administrative areas with the first two considered high resolution); 3) correction for multiple testing: we recorded whether original studies corrected for multiple testing if multiple tests of space-time clustering were performed. We only checked for multiple testing for individual diagnostic groups and not whether studies also accounted for multiple testing across diagnostic groups if different cancer types were analysed; 4) correction for uneven population shifts: we recorded if studies accounted for uneven shifts in the background child population over the study area over the study period.

We developed a quality score based on these four criteria by attributing each study one point per quality criteria met and summing the number of points. The resulting quality index thus ranges from 0 to 4. On the other hand, we did not evaluate the resolution of the time point used in the space-time clustering analysis. In particular, for some of the earlier studies, which obtained case information from death certificates or vital statistics, time of death was treated as time of diagnosis.

In order to minimize the risk of bias across studies, we excluded studies with overlapping data sets from the evidence synthesis and pooled analysis. As a rule, if two studies analysed the same data sample, we included the original study. If a later study analysed a larger sample that fully comprised the sample of an earlier study, we excluded the earlier study. If two studies overlapped only partially, we included both studies. The lists of excluded studies with overlapping data sets are provided below. We also checked whether original studies reported results of clustering tests selectively. To this end we recorded all spatial and temporal lags for which space-time clustering tests were performed according to the methods section and kept track whether results were reported correspondingly. By contrast, we were unable to assess or account for the risk of publication bias in the cumulative evidence because we could not calculate a standardized effect size across original studies to be used in a traditional funnel plot.

## Excluded studies

We excluded studies due to overlapping data sets. Only studies whose sample was completely comprised by another study were excluded. If the overlap was only partial, both studies were included. The following studies were excluded from the evidence synthesis for:

**Leukaemia** for **all ages** combined and age **0-15 years**: Pinkel & Nefzger, 1959 [[31](#_ENREF_31)]; Pinkel et al, 1963 [[32](#_ENREF_32)]; Ederer et al, 1964 [[1](#_ENREF_1)]; Ederer et al, 1965 [[33](#_ENREF_33)]; Gunz & Spears, 1968 [[34](#_ENREF_34)]; Glass & Mantel, 1969 [[35](#_ENREF_35)]; Evatt et al, 1973 [[36](#_ENREF_36)]; Pinder, 1985 [[37](#_ENREF_37)]; Knox & Gilman, 1992 [[38](#_ENREF_38)]; Gilman & Knox, 1995 [[39](#_ENREF_39)]; Gilman et al, 1999 [[40](#_ENREF_40)]; Gustafsson & Carstensen, 1999 [[41](#_ENREF_41)]; McNally et al, 2006 [[42](#_ENREF_42)].

**Leukaemia** for age **0-5 years**: Gunz & Spears, 1968 [[34](#_ENREF_34)]; Knox & Gilman, 1992 [[38](#_ENREF_38)]; Gustafsson & Carstensen, 1999 [[41](#_ENREF_41)]; McNally et al, 2006 [[42](#_ENREF_42)].

**Leukaemia** for age **5-15 years**: Gustafsson & Carstensen, 1999 [[41](#_ENREF_41)]; McNally et al, 2006 [[42](#_ENREF_42)].

**ALL** for **all ages** combined and age **0-15 years**: Pinder, 1985 [[37](#_ENREF_37)]; Gilman et al, 1999 [[40](#_ENREF_40)]; McNally et al, 2006 [[42](#_ENREF_42)].

**ALL** for age **0-5 years** and **5-15 years**: McNally et al, 2006 [[42](#_ENREF_42)].

**Lymphoma**: Mangoud et al, 1985 [[43](#_ENREF_43)]; Knox & Gilman, 1992 [[38](#_ENREF_38)]; Gilman & Knox, 1995 [[39](#_ENREF_39)]; Gilman et al, 1999 [[44](#_ENREF_44)]; Gustafsson & Carstensen, 2000 [[45](#_ENREF_45)]; McNally et al, 2006 [[42](#_ENREF_42)].

**Hodgkin lymphoma**: Mangoud et al, 1985 [[43](#_ENREF_43)]; Gilman et al, 1999 [[44](#_ENREF_44)]; McNally et al, 2006 [[42](#_ENREF_42)].

**Non-Hodgkin lymphoma**: McNally et al, 2006 [[42](#_ENREF_42)].

**Burkitt lymphoma**: Williams et al, 1969 [[46](#_ENREF_46)].

**All CNS tumours** and **astrocytoma**: McNally et al, 2006 [[42](#_ENREF_42)].

**Soft tissue sarcomas**, **renal tumours**, **bone tumours** and **osteosarcomas**: McNally et al, 2006 [[42](#_ENREF_42)].

# References

1. Ederer F, Myers MH, Mantel N. A Statistical Problem in Space and Time: Do Leukemia Cases Come in Clusters? Biometrics. 1964;20(3):626-38. doi:10.2307/2528500

2. Moher D, Liberati A, Tetzlaff J, Altman DG. Preferred Reporting Items for Systematic Reviews and Meta-Analyses: The PRISMA Statement. Annals of Internal Medicine. 2009;151(4):264-9. doi:10.7326/0003-4819-151-4-200908180-00135

3. Fraumeni JF, Jr., Miller RW. Epidemiology of human leukemia: recent observations. J Natl Cancer Inst. 1967;38(4):593-605.

4. Kessler II, Lilienfeld AM. Perspectives in The Epidemiology Of Leukemia*. Advances in Cancer Research. 1969;Volume 12:225-97. doi:<http://dx.doi.org/10.1016/S0065-230X(08)60332-2>

5. Smith P. Spatial and temporal clustering. In: Schottenfeld D, Fraumeni JF, editors. Cancer epidemiology and prevention. Philadelphia: Saunders; 1982. p. 391-407.

6. Linet MS. The leukemias : epidemiologic aspects. New York: Oxford University Press; 1985.

7. Little J. Epidemiology of childhood cancer. Lyon, France: International Agence for Research on Cancer; 1999.

8. McNally RJQ, Eden TOB. An infectious aetiology for childhood acute leukaemia: A review of the evidence. British Journal of Haematology. 2004;127(3):243-63.

9. Chen R, Mantel N, Klingberg MA. A study of three techniques for time-space clustering in hodgkin's disease. Statistics in Medicine. 1984;3(2):173-84. doi:10.1002/sim.4780030210

10. Torabi M, Rosychuk RJ. An examination of five spatial disease clustering methodologies for the identification of childhood cancer clusters in Alberta, Canada. Spatial and Spatio-temporal Epidemiology. 2011;2(4):321-30.

11. Rosychuk RJ, Witol A, Stobart K. Childhood cancer trends in a western Canadian province: A population-based 22-year retrospective study. Pediatric Blood and Cancer. 2010;55(7):1348-55.

12. Thorpe N, Shirmohammadi A. Herbicides and nitrates in groundwater of Maryland and childhood cancers: A geographic information systems approach. Journal of Environmental Science and Health - Part C Environmental Carcinogenesis and Ecotoxicology Reviews. 2005;23(2):261-78.

13. Torabi M, Rosychuk RJ. Hierarchical Bayesian Spatiotemporal Analysis of Childhood Cancer Trends. Geographical Analysis. 2012;44(2):109-20. doi:10.1111/j.1538-4632.2012.00839.x

14. McNally RJQ, Stiller C, Vincent TJ, Murphy MFG. Cross-space-time clustering of childhood cancer in Great Britain: Evidence for a common aetiology. International Journal of Cancer. 2014;134(1):136-43.

15. McNally RJQ, Eden TOB, Alexander FE, Kelsey AM, Birch JM. Is there a common aetiology for certain childhood malignancies? Results of cross-space-time clustering analyses. European Journal of Cancer. 2005;41(18):2911-6.

16. Knox EG, Bartlett MS. The Detection of Space-Time Interactions. Journal of the Royal Statistical Society. Series C (Applied Statistics). 1964;13(1):25-30. doi:10.2307/2985220

17. Knox G. Detection of Low Intensity Epidemicity: Application to Cleft Lip and Palate. British Journal of Preventive & Social Medicine. 1963;17(3):121-7.

18. Bailony MR, Hararah MK, Salhab AR, Ghannam I, Abdeen Z, Ghannam J. Cancer registration and healthcare access in West Bank, Palestine: A GIS analysis of childhood cancer, 1998-2007. International Journal of Cancer. 2011;129(5):1180-9.

19. Miller RW. Deaths from Childhood Cancer in Sibs. New England Journal of Medicine. 1968;279(3):122-6. doi:10.1056/NEJM196807182790302

20. Barnes N, Cartwright RA, O'Brien C, Roberts B, Richards ID, Bird CC. Spatial patterns in electoral wards with high lymphoma incidence in Yorkshire health region. British Journal of Cancer. 1987;56(2):169-72.

21. Mustacchi P. Some Intra-City Variations of Leukemia Incidence in San Francisco. Cancer. 1965;18:362-8.

22. Paldy A, Pinter A, Nador G, Vincze I, Malnasi T. Regional differences of mortality from malignancies in Hungary. [Hungarian] A daganatos halalozas teruleti kulonbsegei Magyarorszagon. Orvosi hetilap. 2003;144(25):1227-33.

23. Barton DE, David FN. The random intersection of two graphs. In: David FN, editor. Research Papers in Statistics (Festschrift for J. Neyman). London: John Wiley & Sons; 1966. p. 445-59.

24. Kuang XF. Epidemiological survey of childhood leukemia. [Chinese]. Zhonghua yu fang yi xue za zhi [Chinese journal of preventive medicine]. 1989;23(4):234-6.

25. Mantel N. The Detection of Disease Clustering and a Generalized Regression Approach. Cancer Research. 1967;27(2 Part 1):209-20.

26. Christiansen T, Lauritsen J, editors. EpiData - Comprehensive Data Management and Basic Statistical Analysis System. Odense, Denmark: EpiData Association; 2010.

27. Diggle PJ, Chetwynd AG, Häggkvist R, Morris SE. Second-order analysis of space-time clustering. Statistical Methods in Medical Research. 1995;4(2):124-36. doi:10.1177/096228029500400203

28. Kulldorff M, Hjalmars U. The Knox method and other tests for space-time interaction. Biometrics. 1999;55(2):544-52.

29. Birch JM, Alexander FE, Blair V, Eden OB, Taylor GM, McNally RJQ. Space-time clustering patterns in childhood leukaemia support a role for infection. British Journal of Cancer. 2000;82(9):1571-6.

30. R Core Team. R: A Language and Environment for Statistical Computing. Vienna, Austria: R Foundation for Statistical Computing; 2017.

31. Pinkel D, Nefzger D. Some epidemiological features of childhood leukemia in the Buffalo, N.Y., area. Cancer. 1959;12(2):351-8. doi:10.1002/1097-0142(195903/04)12:2<351::AID-CNCR2820120218>3.0.CO;2-7

32. Pinkel D, Dowd JE, Bross IDJ. Some epidemiological features of malignant solid tumors of children in the Buffalo, N.Y., area. Cancer. 1963;16(1):28-33. doi:10.1002/1097-0142(196301)16:1<28::AID-CNCR2820160105>3.0.CO;2-U

33. Ederer F, Myers MH, Eisenberg H, Campbell PC. Temporal-Spatial Distribution of Leukemia and Lymphoma in Connecticut. Journal of the National Cancer Institute. 1965;35(4):625-9. doi:10.1093/jnci/35.4.625

34. Gunz FW, Spears GF. Distribution of acute leukaemia in time and space. Studies in New Zealand. British Medical Journal. 1968;4(5631):604-8.

35. Glass AG, Mantel N. Lack of time-space clustering of childhood leukemia in Los Angeles County, 1960-1964. Cancer research. 1969;29(11):1995-2001.

36. Evatt BL, Chase GA, Heath CW. Time-Space Clustering Among Cases of Acute Leukemia in Two Georgia Counties. Blood. 1973;41(2):265-72.

37. Pinder DC. Trends and clusters in leukaemia in Mersey region. Community Medicine. 1985;7(4):272-7.

38. Knox EG, Gilman E. Leukaemia clusters in Great Britain. 1. Space-time interactions. Journal of Epidemiology and Community Health. 1992;46(6):566-72.

39. Gilman EA, Knox EG. Childhood cancers: Space-time distribution in Britain. Journal of Epidemiology and Community Health. 1995;49(2):158-63.

40. Gilman EA, McNally RJ, Cartwright RA. Space-time clustering of acute lymphoblastic leukaemia in parts of the U.K. (1984-1993). European Journal of Cancer. 1999;35(1):91-6.

41. Gustafsson B, Carstensen J. Evidence of space-time clustering of childhood acute lymphoblastic leukaemia in Sweden. British Journal of Cancer. 1999;79(3-4):655-7.

42. McNally RJQ, Alexander FE, Bithell JF. Space-time clustering of childhood cancer in Great Britain: A national study, 1969-1993. International Journal of Cancer. 2006;118(11):2840-6.

43. Mangoud A, Hillier VF, Leck I, Thomas RW. Space-time interaction in Hodgkin's disease in Greater Manchester. Journal of Epidemiology & Community Health. 1985;39(1):58-62.

44. Gilman EA, McNally RJ, Cartwright RA. Space-time clustering of Hodgkin's Disease in parts of the UK, 1984-1993. Leukemia & Lymphoma. 1999;36(1-2):85-100.

45. Gustafsson B, Carstensen J. Space-time clustering of childhood lymphatic leukaemias and non-Hodgkin's lymphomas in Sweden. European Journal of Epidemiology. 2000;16(12):1111-6.

46. Williams EH, Spit P, Pike MC. Further evidence of space-time clustering of Burkitt's lymphoma patients in the West Nile District of Uganda. British journal of cancer. 1969;23(2):235-46.

# Appendix 1: Electronic search strings^[[1]](#footnote-1)^

## MEDLINE

((Neoplasms/

OR

leuk?emia OR acute myeloid leuk?emia OR acute lymphoblastic leuk?emia OR AML OR cancer*1 OR malignan*4 OR cancer cluster*1 OR tumo?r*1 OR CNS OR carcinoma*1 OR glioma*1 OR Hodgkin*2 OR neoplasm*1 OR lymphoma*1 OR blastoma*1 OR sarcoma*1)

AND

(Adolescent/ OR Child/ OR Infant/

OR

child* OR infant*1 OR adolescen*2 OR p?ediatric)

AND

(Space-Time Clustering/ OR Spatial Analysis/ OR Geographic Information Systems/

OR

spatial heterogeneity OR spatial cluster* OR spatial pattern* OR spatio-temporal* OR time-space OR space-time OR Cuzick-Edwards OR K-function* OR Knox* OR close pair* OR Moran* OR Mantel OR Potthoff-Witthinghill OR Rogerson* OR kernel OR scan statistic*1 OR spatial scan* OR SaTScan OR FleXScan))

## Embase

((neoplasm/

OR

leuk?emia OR acute myeloid leuk?emia OR acute lymphoblastic leuk?emia OR AML OR cancer*1 OR malignan*4 OR cancer cluster*1 OR tumo?r*1 OR CNS OR carcinoma*1 OR glioma*1 OR Hodgkin*2 OR neoplasm*1 OR lymphoma*1 OR blastoma*1 OR sarcoma*1)

AND

(adolescent/ OR child/ OR infant/

OR

child* OR infant*1 OR adolescen*2 OR p?ediatric)

AND

(space-time clustering/ OR spatial analysis/ OR geographic information systems/

OR

spatial heterogeneity OR spatial cluster* OR spatial pattern* OR spatio-temporal* OR time-space OR space-time OR Cuzick-Edwards OR K-function* OR Knox* OR close pair* OR Moran* OR Mantel OR Potthoff-Witthinghill OR Rogerson* OR kernel OR scan statistic*1 OR spatial scan* OR SaTScan OR FleXScan))

# Appendix 2: Data items extracted

The following data items were extracted from included studies. Terms in brackets [] indicate variable names in the annexed data files.

## Systematic Review

- Publication
  - Unique ID [ID]
  - First author [Author]
  - Year of publication [Year]
  - Title [Title]
  - Journal/book section [Journal]
  - Volume [Volume]
  - Issue [Issue]
  - Pages [Pages]
- Data
  - Study region [m1]
  - Cancer registry/study population [m2]
  - Cases [m3]
  - Source population (census data for case-control studies and regional count data) [m4]
  - Study design (case only [1], case-control [2], or regional count[3]) [m5]
  - Diagnostic groups (all cancers [d1], leukaemia [d2], lymphomas [d3], CNS tumors [d4], and other cancer types [d5])
  - Spatial resolution of address information (e.g. exact geocodes, postcodes, census tracts, health service areas, municipalities) [r1]
  - Study period (begin [sp1], end [sp2], and study intervals if applicable [sp3])
  - Age range of cases [a1] and analyses by age subgroups [a2]
  - Place and time point of clustering analyses (i.e. place of residence at birth or diagnosis/death combined with date of birth or diagnosis/death: B*dob [stc1], B*dox [stc2], X*dob [stc3], X*dox [stc4])
- Methodology
  - Statistical tests: general (e.g. Mantel, Knox, K-functions) or focused (e.g. SatScan, FlexScan) [stc5]
  - Null distribution (e.g. Poisson, Normal, Monte Carlo simulation) [stc6]
  - Spatial and temporal lags of clustering tests [stc7; stc8]
  - Correction for multiple testing [stc9]
  - Adjustment for shifts in the background (child) population [stc10]
- Results
  - Evidence of clustering by cancer type, age group and computation method around place and time of birth [rsct1; rsct1a] or diagnosis [rsct2; rsct2a], including spatial and temporal lags that maximize the evidence of clustering
  - Indicator of strength of clustering (e.g. the excess number of close pairs observed over the number expected under the assumption of no clustering, p-value of test etc.) [cr]
- Remarks
  - Study population [cp]
  - Methodology [cm]

## Meta-analysis of studies using Knox tests

- - Diagnostic group [ICCC3]
  - Age range [Age]
  - Study period [Period]
  - Spatial lag [Kilometers]
  - Temporal lag [Months]
  - Number of observed close pairs [Obs]
  - Number of expected close pairs under the assumption of no space-time interaction [Exp]
  - Strength of clustering (i.e. number of observed close pairs in excess of the number expected) [Strength %]
  - Variance, standard deviation, or p-value [SD]

1. We removed the terms “ALL”, “cALL”, “aetiology”, “infections” and “viral hypothesis” from the final search terms for neoplasms as these proved too vague, resulting in an unmanageable amount of publications. Notably the terms “ALL” and “cALL” hugely inflated the number of hits due to the impossibility of Ovid to distinguish upper and lower case letters. We removed the terms “spatial distribution” and “geographical distribution” from the list of GIS/clustering terms as these too proved to be too vague and resulted in an unmanageable number of publications. [↑](#footnote-ref-1)
