# Supplementary material for: Space–time clustering of childhood cancers: a systematic review and pooled analysis
Source: Eur J Epidemiol. 2018 Nov 16;34(1):9–21. doi: 10.1007/s10654-018-0456-y (PMC6326085; doi:10.1007/s10654-018-0456-y)
Supplement: Supplementary file 2 — Supplementary material 2 (PDF 1657 kb) [file 10654_2018_456_MOESM2_ESM.pdf]

Figure S1 Pooled analysis of space-time clustering studies of childhood **leukaemia** for place and time of **diagnosis** for children aged **0-15 years** (top), **0-5 years** (middle) and **5-15 years** (bottom): Forrest plot of ratio of observed over expected number of close pairs of cases (O/E) and acceptance region for one-sided Knox test assuming Poisson distribution at 5% alpha-level

Author, Year	Country	Period	Diagnosis	Age	km	mths	O/E	P-value	Weight
Meighan et al., 1965	USA	1950-61	Leuk	0-14	8	8	1.14	0.05	0.7%
Mainwaring, 1966	UK	1955-64	AL	0-14	8	8	1.04	0.22	1.3%
Till et al., 1967	UK	1952-60	LL	0-14	1	2	1.96	0.02	0%
Gunz et al., 1968	NZ	1953-64	Leuk	0-14	8	3	1.17	0.18	0.1%
Glass et al., 1969	USA	1960-64	Leuk	0-14	7	6	0.98	0.58	0.9%
Zahálková et al., 1970	CZ	1965-68	Leuk	0-15	12	12	1.88	0.04	0%
Klauber et al., 1970	USA	1946-66	Leuk	0-14	3	12	1.04	0.23	1.3%
Van Steensel-Moll et al., 1983	NL	1973-79	Leuk	0-14	2	6	1.02	0.41	0.2%
Morris, 1990	UK	1953-60	Leuk	0-9	0	15	1.92	0.02	0%
Gilman et al., 1991	UK	1966-83	Leuk	0-14	5	9	1.01	0.21	31.8%
Petridou et al., 1996	GR	1980-89	Leuk	0-14	5	12	1.05	<0.01	14.1%
Gustafsson et al., 1999	SWE	1973-89	ALL	0-15	25	6	0.99	0.51	0.9%
Gilman et al., 1999	UK	1984-88	ALL	0-14	5	9	0.99	0.5	0.4%
Gilman et al., 1999	UK	1989-93	ALL	0-14	5	9	0.98	0.56	0.5%
Bellec et al., 2006	F	1990-00	AL	0-14	5	9	1	0.5	45.9%
Kreis et al., 2016	CH	1985-10	Leuk	0-15	5	6	0.91	0.96	1.9%
					**6.5**	**8.3**	**1.01**	**0.05**	**100%**

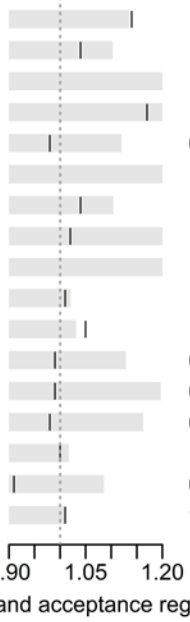

Author, Year	Country	Period	Diagnosis	Age	km	mths	O/E	P-value	Weight
Knox, 1964	UK	1951-60	Leuk	0-5	1	2	6.25	<0.01	0%
Till et al., 1967	UK	1952-60	LL	0-5	1	2	2.22	0.02	0.1%
Gunz et al., 1968	NZ	1953-64	Leuk	0-5	8	3	1.14	0.27	0.2%
Browning et al., 1968	USA	1952-60	AL	0-5	4	3	1.05	0.33	0.1%
Van Steensel-Moll et al., 1983	NL	1973-79	ALL	0-5	2	6	0.61	0.86	0.2%
Gilman et al., 1991	UK	1966-83	Leuk	0-4	5	6	1.03	0.16	25.5%
Petridou et al., 1996	GR	1980-89	Leuk	0-4	5	12	1.09	<0.01	18.4%
Gustafsson et al., 1999	SWE	1973-89	ALL	0-4	25	6	1.03	0.36	1.8%
Birch et al., 2000	UK	1954-85	Leuk	0-4	5	12	1.11	0.03	7.2%
Bellec et al., 2006	F	1990-00	AL	0-4	5	6	1.04	0.04	44.2%
Kreis et al., 2016	CH	1985-10	Leuk	0-4	5	6	1.01	0.43	2.3%
					**6**	**5.9**	**1.05**	**<0.01**	**100%**

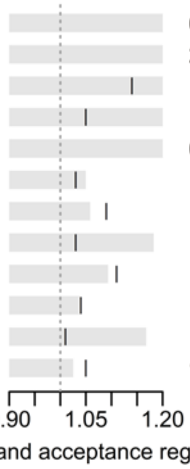

Author, Year	Country	Period	Diagnosis	Age	km	mths	O/E	P-value	Weight
Gilman et al., 1991	UK	1966-83	Leuk	5-14	10	12	0.99	0.78	93.1%
Gustafsson et al., 1999	SWE	1973-89	ALL	5-15	25	12	0.98	0.5	0.6%
Kreis et al., 2016	CH	1985-10	Leuk	5-15	10	12	0.85	1	6.4%
					**15**	**12**	**0.98**	**0.94**	**100%**

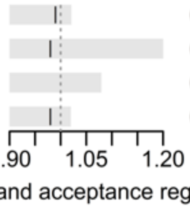

Birch et al, 2000, Gilman & Knox, 1995, Knox & Gilman, 1992 and Pinder, 1985 were excluded from the analysis of children aged 0-15 years because of overlapping study samples.

Figure S2 Pooled analysis of space-time clustering studies of childhood **leukaemia** for place and time of **birth** for children aged **0-15 years** (top), **0-5 years** (middle) and **5-15 years** (bottom): Forrest plot of ratio of observed over expected number of close pairs of cases (O/E) and acceptance region for one-sided Knox test assuming Poisson distribution at 5% alpha-level

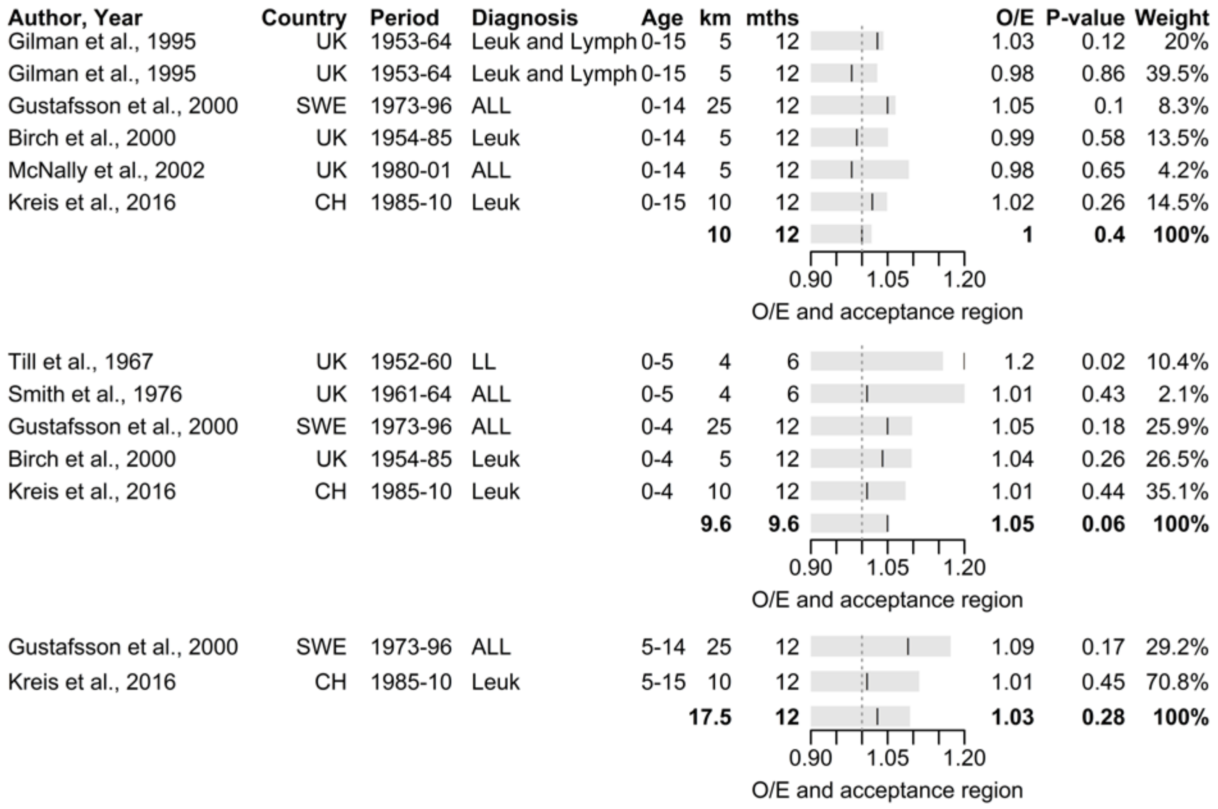

Morris, 1990 was excluded from the analysis of children aged 0-15 years because of overlapping study samples. Smith et al, 1976 (sub-period 1952-59) was excluded from the analysis of children aged 0-5 years because of overlapping study samples.

Figure S3 Pooled analysis of space-time clustering studies of childhood **leukaemia** for place and time of **diagnosis** for children aged **0-15 years** (top) and **0-5 years** (bottom) for spatial lags of **3-7 km** and temporal lags of **6-18 months**: Forrest plot of ratio of observed over expected number of close pairs of cases (O/E) and acceptance region for one-sided Knox test assuming Poisson distribution at 5% alpha-level

Author, Year	Country	Period	Diagnosis	Age	km	mths	O/E	P-value	Weight
Meighan et al., 1965	USA	1950-61	Leuk	0-14	4	12	1.18	0.06	0.5%
Mainwaring, 1966	UK	1955-64	AL	0-14	5	12	1.15	0.02	1.5%
Glass et al., 1969	USA	1960-64	Leuk	0-14	4	12	0.95	0.66	0.6%
Klauber et al., 1970	USA	1946-66	Leuk	0-14	3	12	1.04	0.23	1.8%
Gilman et al., 1991	UK	1966-83	Leuk	0-14	5	12	1.01	0.18	59.6%
Petridou et al., 1996	GR	1980-89	Leuk	0-14	5	12	1.05	<0.01	20.3%
Gilman et al., 1999	UK	1984-88	ALL	0-14	5	12	0.98	0.55	0.7%
Gilman et al., 1999	UK	1989-93	ALL	0-14	5	12	1.01	0.43	1%
Bellec et al., 2006	F	1990-00	AL	0-14	5	12	1	0.49	8.6%
Kreis et al., 2016	CH	1985-10	Leuk	0-15	5	12	0.94	0.96	5.3%
					**4.6**	**12**	**1.02**	**0.02**	**100%**

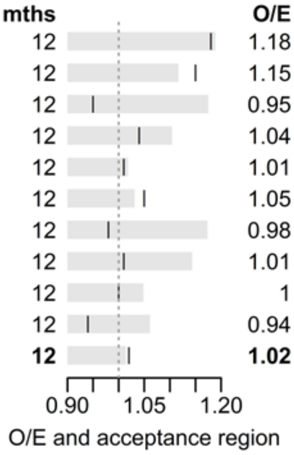

Author, Year	Country	Period	Diagnosis	Age	km	mths	O/E	P-value	Weight
Gilman et al., 1991	UK	1966-83	Leuk	0-4	5	12	1.02	0.18	29.8%
Petridou et al., 1996	GR	1980-89	Leuk	0-4	5	12	1.09	<0.01	11.4%
Birch et al., 2000	UK	1954-85	Leuk	0-4	5	12	1.11	0.03	4.4%
Bellec et al., 2006	F	1990-00	AL	0-4	5	12	1.01	0.27	51.5%
Kreis et al., 2016	CH	1985-10	Leuk	0-4	5	12	1.02	0.38	2.9%
					**5**	**12**	**1.03**	**0.01**	**100%**

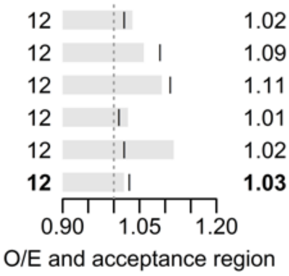

Birch et al, 2000, Gilman & Knox, 1995, Knox & Gilman, 1992 and Pinder, 1985 were excluded from the analysis of children aged 0-15 years because of overlapping study samples.

Figure S4 Pooled analysis of space-time clustering studies of childhood **leukaemia** for place and time of **diagnosis** for children aged **0-15 years** (top) and **0-5 years** (bottom) for spatial lags of **3-7 km** and temporal lags of **12-36 months**: Forrest plot of ratio of observed over expected number of close pairs of cases (O/E) and acceptance region for one-sided Knox test assuming Poisson distribution at 5% alpha-level

Author, Year	Country	Period	Diagnosis	Age	km	mths	O/E	P-value	Weight
Meighan et al., 1965	USA	1950-61	Leuk	0-14	4	12	1.18	0.06	0.3%
Mainwaring, 1966	UK	1955-64	AL	0-14	5	33	1.09	0.02	2.4%
Glass et al., 1969	USA	1960-64	Leuk	0-14	4	12	0.95	0.66	0.4%
Klauber et al., 1970	USA	1946-66	Leuk	0-14	3	12	1.04	0.23	1.1%
Gilman et al., 1991	UK	1966-83	Leuk	0-14	5	24	1.01	0.1	70.3%
Petridou et al., 1996	GR	1980-89	Leuk	0-14	5	12	1.05	<0.01	12.4%
Gilman et al., 1999	UK	1984-88	ALL	0-14	5	18	0.97	0.61	0.6%
Gilman et al., 1999	UK	1989-93	ALL	0-14	5	18	1.05	0.23	0.8%
Bellec et al., 2006	F	1990-00	AL	0-14	5	12	1	0.49	5.2%
Kreis et al., 2016	CH	1985-10	Leuk	0-15	5	24	0.97	0.9	6.4%
					**4.6**	**17.8**	**1.01**	**0.02**	**100%**

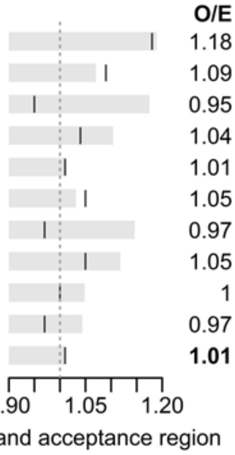

Author, Year	Country	Period	Diagnosis	Age	km	mths	O/E	P-value	Weight
Gilman et al., 1991	UK	1966-83	Leuk	0-4	5	24	1.01	0.26	43.7%
Petridou et al., 1996	GR	1980-89	Leuk	0-4	5	12	1.09	<0.01	8.8%
Birch et al., 2000	UK	1954-85	Leuk	0-4	5	12	1.11	0.03	3.4%
Bellec et al., 2006	F	1990-00	AL	0-4	5	12	1.01	0.27	39.7%
Kreis et al., 2016	CH	1985-10	Leuk	0-4	5	24	0.97	0.72	4.4%
					**5**	**16.8**	**1.02**	**0.04**	**100%**

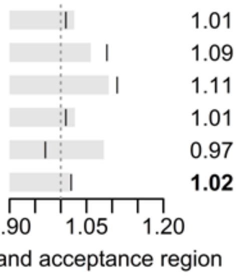

Birch et al, 2000, Gilman & Knox, 1995, Knox & Gilman, 1992 and Pinder, 1985 were excluded from the analysis of children aged 0-15 years because of overlapping study samples.

Figure S5 Pooled analysis of space-time clustering studies of childhood **leukaemia** for place and time of **diagnosis** for children aged **0-15 years** (top) and **0-5 years** (bottom) for spatial lags of **7-15 km** and temporal lags of **0-12 months**: Forrest plot of ratio of observed over expected number of close pairs of cases (O/E) and acceptance region for one-sided Knox test assuming Poisson distribution at 5% alpha-level

Author, Year	Country	Period	Diagnosis	Age	km	mths	O/E	P-value	Weight
Meighan et al., 1965	USA	1950-61	Leuk	0-14	8	7	1.12	0.09	0.3%
Mainwaring, 1966	UK	1955-64	AL	0-14	8	7	1.01	0.43	0.6%
Gunz et al., 1968	NZ	1953-64	Leuk	0-14	8	3	1.17	0.18	0.1%
Glass et al., 1969	USA	1960-64	Leuk	0-14	11	6	1	0.46	1.2%
Zahálková et al., 1970	CZ	1965-68	Leuk	0-15	12	12	1.88	0.04	0%
Gilman et al., 1991	UK	1966-83	Leuk	0-14	10	6	1.01	0.12	41.5%
Bellec et al., 2006	F	1990-00	AL	0-14	10	6	1	0.5	53.3%
Kreis et al., 2016	CH	1985-10	Leuk	0-15	10	6	0.95	0.92	2.9%
					**9.6**	**6.5**	**1**	**0.26**	**100%**

Author, Year	Country	Period	Diagnosis	Age	km	mths	O/E	P-value	Weight
Gunz et al., 1968	NZ	1953-64	Leuk	0-5	8	3	1.14	0.27	0.1%
Gilman et al., 1991	UK	1966-83	Leuk	0-4	10	6	1.02	0.12	39.7%
Bellec et al., 2006	F	1990-00	AL	0-4	10	6	1.02	0.08	57.4%
Kreis et al., 2016	CH	1985-10	Leuk	0-4	10	6	1.03	0.29	2.8%
					**9.5**	**5.2**	**1.02**	**0.03**	**100%**

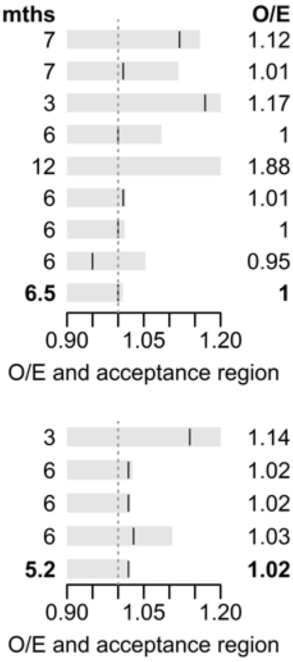

Birch et al, 2000, Gilman & Knox, 1995, Knox & Gilman, 1992 and Pinder, 1985 were excluded from the analysis of children aged 0-15 years because of overlapping study samples.

Figure S6 Pooled analysis of space-time clustering studies of childhood **ALL** for place and time of **diagnosis** for children aged **0-15 years** (top), **0-5 years** (middle) and **5-15 years** (bottom): Forrest plot of ratio of observed over expected number of close pairs of cases (O/E) and acceptance region for one-sided Knox test assuming Poisson distribution at 5% alpha-level

Author, Year	Country	Period	Diagnosis	Age	km	mths	O/E	P-value	Weight
Till et al., 1967	UK	1952-60	LL	0-14	1	2	1.96	0.02	0.1%
Van Steensel-Moll et al., 1983	NL	1973-79	ALL	0-14	2	6	1.01	0.43	0.4%
Gilman et al., 1991	UK	1966-83	LL	0-14	5	9	0.99	0.73	58.7%
Petridou et al., 1996	GR	1980-89	ALL	0-14	5	12	1.04	0.04	31.7%
Gustafsson et al., 1999	SWE	1973-89	ALL	0-15	25	6	0.99	0.51	2.7%
Gilman et al., 1999	UK	1984-88	ALL	0-14	5	9	0.99	0.5	1.1%
Gilman et al., 1999	UK	1989-93	ALL	0-14	5	9	0.98	0.56	1.6%
Kreis et al., 2016	CH	1985-10	ALL	0-15	5	6	0.94	0.81	3.7%
					**6.6**	**7.4**	**1**	**0.36**	**100%**

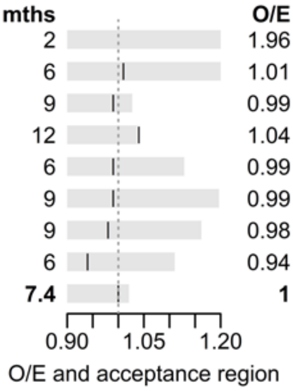

Author, Year	Country	Period	Diagnosis	Age	km	mths	O/E	P-value	Weight
Till et al., 1967	UK	1952-60	LL	0-5	1	2	2.22	0.02	0.2%
Van Steensel-Moll et al., 1983	NL	1973-79	ALL	0-5	2	6	0.61	0.86	0.8%
Petridou et al., 1996	GR	1980-89	ALL	0-4	5	12	1.05	0.1	52.8%
Gustafsson et al., 1999	SWE	1973-89	ALL	0-4	25	12	1.07	0.17	13.3%
Birch et al., 2000	UK	1954-85	ALL	0-4	5	12	1.1	0.05	20.5%
Kreis et al., 2016	CH	1985-10	ALL	0-4	5	12	0.97	0.64	12.3%
					**7.1**	**9.4**	**1.05**	**0.04**	**100%**

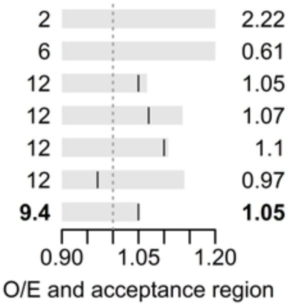

Author, Year	Country	Period	Diagnosis	Age	km	mths	O/E	P-value	Weight
Gustafsson et al., 1999	SWE	1973-89	ALL	5-15	25	12	0.98	0.5	13.5%
Kreis et al., 2016	CH	1985-10	ALL	5-15	10	12	0.85	0.99	86.5%
					**17.5**	**12**	**0.87**	**0.99**	**100%**

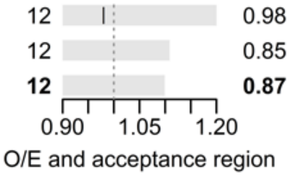

Birch et al, 2000, Gilman & Knox, 1995, Knox & Gilman, 1992 and Pinder, 1985 were excluded from the analysis of children aged 0-15 years because of overlapping study samples.

Figure S7 Pooled analysis of space-time clustering studies of childhood **ALL** for place and time of **birth** for children aged **0-15 years** (top), **0-5 years** (middle) and **5-15 years** (bottom): Forrest plot of ratio of observed over expected number of close pairs of cases (O/E) and acceptance region for one-sided Knox test assuming Poisson distribution at 5% alpha-level

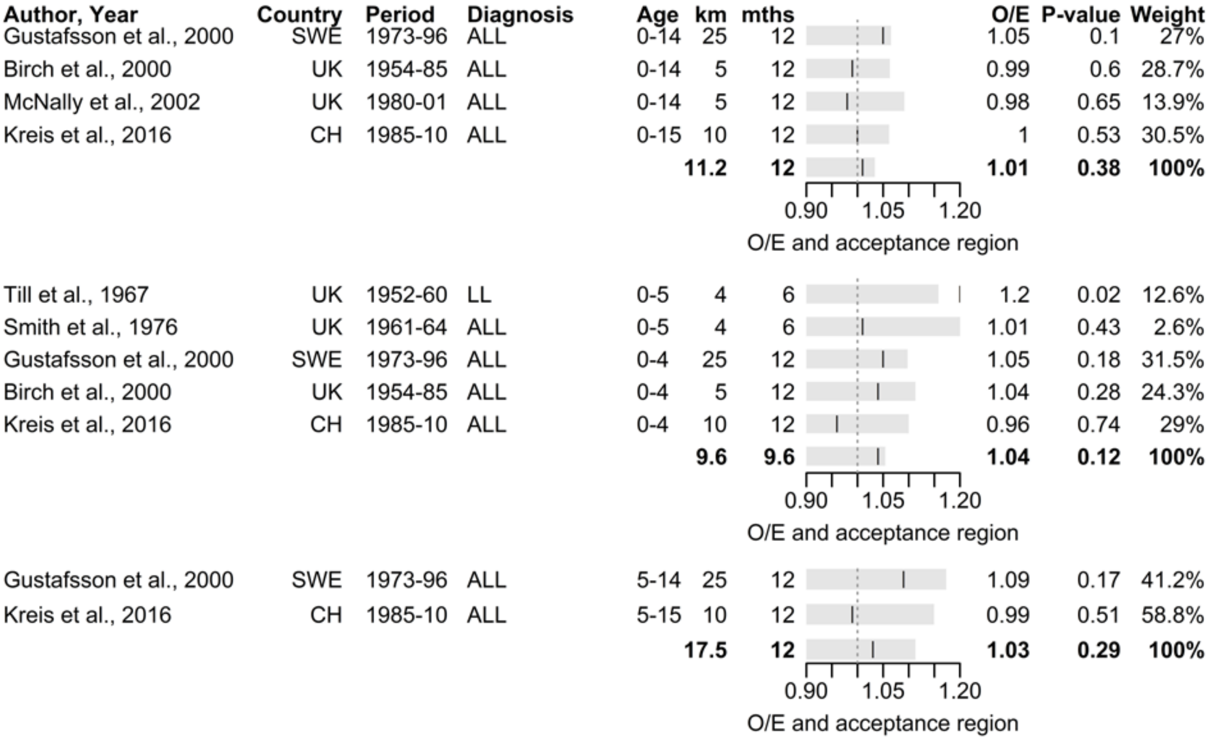

Figure S8 Pooled analysis of space-time clustering studies of childhood **lymphoma** for place and time of **diagnosis** (top) and **birth** (upper middle), **CNS tumours** for place and time of **diagnosis** (lower middle) and for **neuroblastomas** for place and time of **birth** (bottom): Forrest plot of ratio of observed over expected number of close pairs of cases (O/E) and acceptance region for one-sided Knox test assuming Poisson distribution at 5% alpha-level

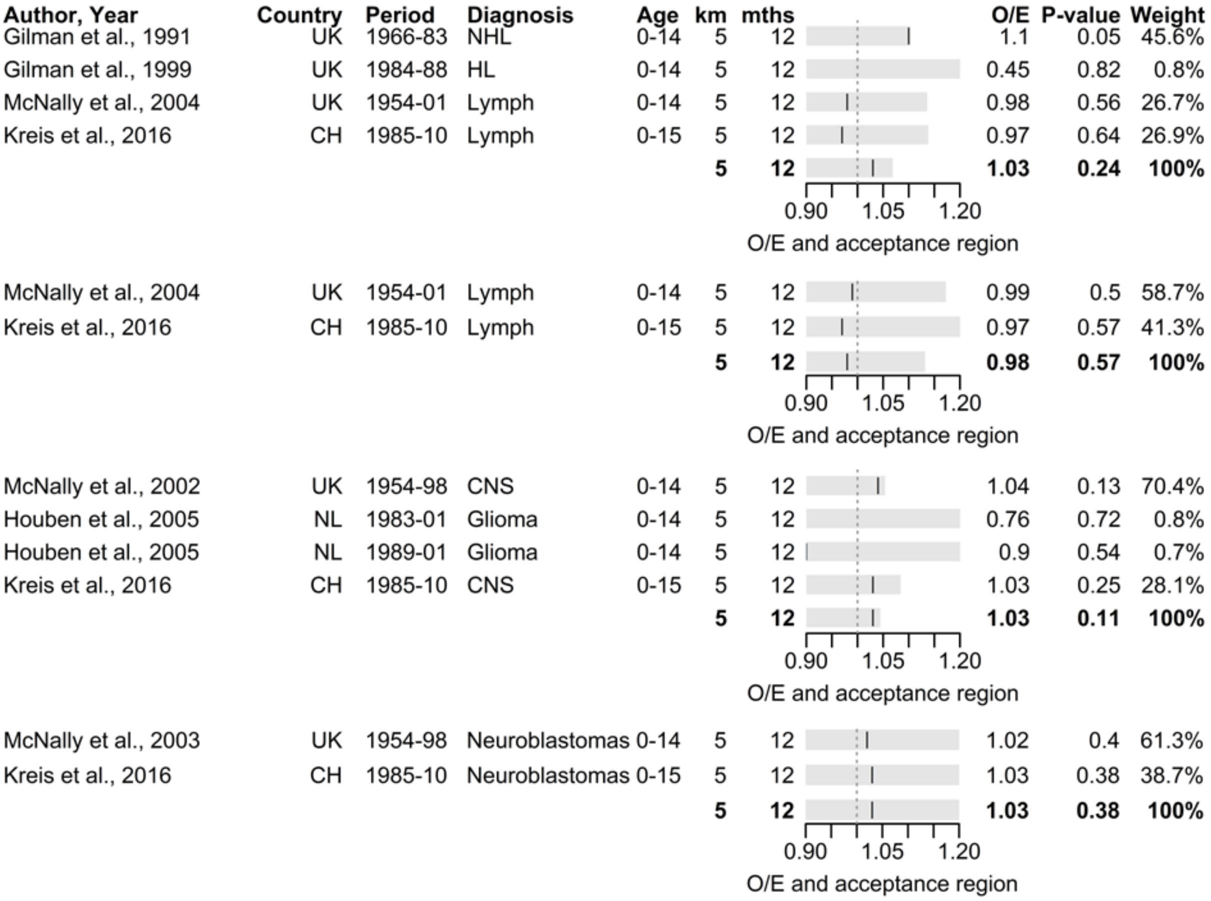

Mangoud et al, 1985 was excluded from the analysis of childhood lymphoma at time of diagnosis because of overlapping study samples.
