# Supplementary material for: Space–time clustering of childhood cancers: a systematic review and pooled analysis
Source: Eur J Epidemiol. 2018 Nov 16;34(1):9–21. doi: 10.1007/s10654-018-0456-y (PMC6326085; doi:10.1007/s10654-018-0456-y)
Supplement: Supplementary file 3 — Supplementary material 3 (PDF 137 kb) [file 10654_2018_456_MOESM3_ESM.pdf]

Table S1 Space-time clustering studies of childhood leukaemia

Author, Year	Study area	Period	Quality	Resolution	Critical lags	Cancer type	Age	Time	Cases	Method	Results		
											Rate of sig. Tests[a]		Significant test[b]
Pinkel et al., 1959	Buffalo Area, NY, USA	1943-1956	2	Postcodes	s: 1/3 mile<br>t: 2 years	Leuk	0-15	X	137	Combinatorial analysis	0	(0/2)	n.s.
Pinkel et al., 1963	Buffalo Area, NY, USA	1943-1956	2	Postcodes	s: 1/8, 2/8, ..., 1 mile<br>t: not specified	Leuk	0-15	X	206	Average ridit	0.125	(1/8)	Leuk, 0-15y, 1/8m
Ederer et al., 1964	State of Connecticut, USA	1945-1959	1	Areas	s: same town<br>t: 1, 2 years	Leuk	0-14	X	333	E-M-M	0	(0/2)	n.s.
Knox, 1964	Northumberland and Durham, UK	1951-1960	1	Areas	s: 1, >1 km<br>t: 2 months	Leuk	0-14	X	185	Knox	0	(0/NA)	Leuk, 0-5y, 1km, 2mths
						ML	0-14	X	46	Knox	0	(0/NA)	
						LL	6-14	X	50	Knox	0	(0/NA)	
						Leuk	0-5	X	96	Knox	NA	(1/NA)	
Meighan et al., 1965	Portland, Oregon, USA	1950-1961	1	Geocodes	s: 1, 2, 4, 8 km<br>t: 50, 100, ..., 300, 365 days	Leuk	0-14	X	69	Knox	0.071	(2/28)	Leuk, 0-14y, 4km, 250d
Ederer et al., 1965	State of Connecticut, USA	1945-1959	2	Areas	s: same town<br>t: 1, 2 years	Leuk	0-14	X	448	E-M-M	0	(0/2)	n.s.
Fraumeni et al., 1966	Upper New York State, USA	1943-1962	1	Areas	s: same town<br>t: 1, 2 years	Leuk	0-14	X	1640	E-M-M	1	(2/2)	Leuk, 0-14y, same t., 1y; Leuk, 0-4y, same t., 2y
						Leuk	0-9	X	1368	E-M-M	0	(0/2)	
						Leuk	0-4	X	816	E-M-M	1	(2/2)	
Mainwaring, 1966	Liverpool Area, UK	1955-1964	1	Geocodes	s: 2, 3, 4, 5, 8 km<br>t: 50, 100, ..., 400, 1000 days	AL	0-14	X	74	Knox	0.2	(9/45)	AL, 0-14y, 4km, 300d
						AL	0-5	X	37	Knox	NA	(NA/NA)	
						AL	6-14	X	37	Knox	NA	(NA/NA)	
Stark et al., 1967	State of Michigan, USA	1950-1963	0	Areas	s: state; same county<br>t: 3, 4 years	Leuk	0-4	B	375	E-M-M	0	(0/1)	n.s.
						Leuk	0-1	B	77	E-M-M	0	(0/1)	
Till et al., 1967	Greater London, UK	1952-1960	2	Geocodes	s: 0.25, 0.5, ..., 2, 2.5, ..., 4 km<br>t: 0.5, 1, ..., 6 months	Leuk	0-14	X	444	Knox	0	(0/144)	B: Knox: LL, 0-5y, 3.5km, 6mths<br>X: Knox: LL, 0-14y, 0.75km, 2.5mths
						Leuk	0-5	X	315	Knox	0	(0/144)	
						LL	0-14	X	346	Knox	0.007	(1/144)	
						LL	0-5	X	252	Knox	0	(0/144)	
						Leuk	0-5	B	292	Knox	0	(0/144)	
						LL	0-5	B	232	Knox	0.056	(8/144)	
						Leuk	0-14	X	444	David & Barton 1966	0	(0/144)	
						Leuk	0-5	X	315	David & Barton 1966	0	(0/144)	
						LL	0-14	X	346	David & Barton 1966	0	(0/144)	
						LL	0-5	X	252	David & Barton 1966	0	(0/144)	
Gunz et al., 1968	New Zealand	1953-1964	1	Postcodes	s: 1, 5, 10 miles<br>t: 2, 3, 6 months	Leuk	0-14	X	288	Knox	0	(0/4)	Leuk, 0-5y, 1m, 2mths
						Leuk	0-5	X	160	Knox	0.5	(2/4)	

Table S1 continued

Author, Year	Study area	Period	Quality	Resolution	Critical lags	Cancer type	Age	Time	Cases	Method	Results		
											Rate of sig. Tests[a]		Significant test[b]
Browning et al., 1968	Cuyahoga County, Ohio, USA	1955-1965	0	Areas	s: 1, 2, 4 km<br>t: 60, 90 days	AL	0-14	X	168	Knox	0	(0/6)	n.s.
Glass et al., 1968	Los Angeles County, USA	1960-1964	1	Geocodes	s: same census region<br>t: 1 year	Leuk	0-14	X	298	E-M-M	0	(0/1)	n.s.
Klauber, 1968	State of California, USA	1958-1960	1	Areas	s: same hospital<br>t: 1, 3, 5, 15, 30 days	Leuk	0-4	B	NA	Mantel	0	(0/5)	n.s.
Glass et al., 1969	Los Angeles County, USA	1960-1964	1	Postcodes	s: Knox: 0.53-13.2 km; M: 0.09-6.6 km<br>t: 0.5, 1, 2, 4, 6, 12 months	Leuk	0-14	X	298	Knox	0	(0/48)	n.s.
						Leuk	0-5	X	155	Knox	0	(0/48)	
						Leuk	2-9	X	205	Knox	0	(0/48)	
						Leuk	0-14	X	298	Mantel	0	(0/60)	
						Leuk	0-5	X	155	Mantel	0	(0/60)	
						Leuk	2-9	X	205	Mantel	0	(0/60)	
Klauber et al., 1970	San Francisco, USA	1946-1966	2	Geocodes	s: M: average distance; Knox: 0.25, 0.5, 1, 2 miles<br>t: M: 0.5, 1, 2, 4, 12 months; K: 30, 60, 120, 365 days	Leuk	0-14	X	149	Mantel	0	(0/5)[1]	M: Leuk, 2-14y, 12mths
						Leuk	2-14	X	121	Mantel	0.2	(1/5)[1]	
Bailar et al., 1970	State of Connecticut, USA	1935-1963	0	Areas	s: same town<br>t: 1, 2, ..., 28 years	Leuk	0-14	X	4552	E-M-M (modified)	0	(0/NA)	n.s.
Zahálková et al., 1970	South Moravian Province, Czech Republic	1960-1968	0	Areas	s: 10, 12, 15 km<br>t: 1, 2 years	Leuk	0-15	X	104	Knox	0.5	(3/6)	Leuk, 0-15y, 12km, 2y
Glass et al., 1971	New Zealand	1952-1963	2	Geocodes	s: 0.5, 1, 2, 3, 5 miles<br>t: 0.5, 1, 2, 3, 6 months	AL*	0-14	X	288	Knox	0.56	(14/25)	Knox: AL, 0-14y, 1m, 1mth; AL, 0-5y, 1m, 0.5mth; AL, 2-9y, 0.5m, 1mth; M: AL, 0-14y, 0.5m, 0.5mth; AL, 0-5y, 0.5m, 0.5mth; AL, 2-9y, 0.5m, 0.5mth
						AL*	0-5	X	160	Knox	0.68	(17/25)	
						AL*	2-9	X	202	Knox	0.24	(6/25)	
						AL*	0-14	X	288	Mantel	0.96	(24/25)	
						AL*	0-5	X	160	Mantel	0.48	(12/25)	
						AL*	2-9	X	202	Mantel	0.36	(9/25)	
Evatt et al., 1973	DeKalb and Fulton Counties, Georgia, USA	1956-1968	2	Areas	s: same census tract<br>t: 2, 3 years	AL	0-14	X	96	Pearsons Chi-square, Haldanes moments	0.5	(2/4)[2]	AL, 0-14, 2y, DeKalb, 56-67; AL, 0-14, 2y, Fulton, 56-67; AL, 0-14, 2y, both, 56-67
Larsen et al., 1973	Metropolitan Atlanta, Georgia, USA	1956-1969	2	Areas	s: same census tract<br>t: 6 months	AL	0-14	X	164	K statistic	1	(1/1)	K statistic: AL, 0-14y, 6 census tracts
						AL	0-14	X	164	Knox	0	(0/NA)	
						AL	0-14	X	164	Mantel	0	(0/NA)	
						ALL	0-14	X	124	K statistic	1	(1/1)	
						ALL	0-14	X	124	Knox	0	(0/NA)	
						ALL	0-14	X	124	Mantel	0	(0/NA)	

Table S1 continued

Author, Year	Study area	Period	Quality	Resolution	Critical lags	Cancer type	Age	Time	Cases	Method	Results		
											Rate of sig. Tests[a]		Significant test[b]
						AL**	0-14	X	23	K statistic	0	(0/1)	
						AL**	0-14	X	23	Knox	0	(0/NA)	
						AL**	0-14	X	23	Mantel	0	(0/NA)	
Smith et al., 1976	Greater London, UK	1952-1965	3	Geocodes	s: 0.25, 0.5, ..., 2, 2.5, ..., 4 km t: 0.5, 1, ..., 6 months	ALL	0-5	B	172	Knox	0.028	(4/144)[3]	ALL, 0-5y, 1.75km, 4mths, 52-59
						ALL	0-5	B	81	Knox	0	(0/144)[4]	
Van Steensel-Moll et al., 1983	Western Part of the Netherlands	1973-1980	1	Areas	s: 2, 4, 6, 8, 10 km t: 2, 4, 6, 8, 10, 12 months	Leuk	0-14	X	293	Mantel	0	(0/1)	ALL, 0-14y, 2km, 6mths (only ALL patients with >50'000 leucocytes/mm3)
						ALL	0-14	X	233	Mantel	0	(0/1)	
						ALL	0-5	X	159	Mantel	0	(0/1)	
						ANLL	0-14	X	49	Mantel	0	(0/1)	
						Leuk	0-14	X	293	Knox	0	(0/30)	
						ALL	0-14	X	233	Knox	0	(0/30)	
						ALL	0-5	X	159	Knox	0	(0/30)	
						ANLL	0-14	X	49	Knox	0	(0/30)	
Pinder, 1985	Mersey Region, UK	1974-1983	2	Postcodes	s: 0.5, 1, 2, 4, >=4 km t: 1, 2, 4, 8, 12, 24, 48, >48 months	Leuk	0-14	X	165	X2	0	(0/1)	n.s.
						Leuk	0-6	X	85	X2	0	(0/1)	
						LL	0-14	X	125	X2	0	(0/1)	
						LL	0-6	X	65	X2	0	(0/1)	
Morris, 1990	Worcestershire, Warwickshire, Staffordshire, Shropshire; UK	1953-1960	2	Geocodes	s: not specified t: not specified	Leuk	0-6	B	162	Knox	NA	(1/NA)	B: Leuk, 0-6y, 2km, 248d X: Leuk, 0-9y, 0.5km, 15mths
						Leuk	0-9	X	228	Knox	NA	(1/NA)	
Gilman et al., 1991	England, Scotland and Wales, UK	1966-1983	3	Postcodes	s: 1, 2, 3, 4, 5, 10, 20 km t: 0, 1, ..., 9, 12, 18, 24, 48 months	LL	0-14	X	5710	Knox	0.036	(3/84)[5]	LL, 0-14y, 1km, 0mths; ANLL, 0-14y, 2km, 5mths; Leuk, 0-14y, 1km, 3mths; Leuk, 0-4y, 1km, 9m
						ANLL	0-14	X	1241	Knox	0.06	(5/84)[5]	
						LL*	0-14	X	6094	Knox	0.024	(2/84)[5]	
						Leuk	0-14	X	7565	Knox	0.024	(2/84)[5]	
						Leuk	0-4	X	3856	Knox	0.06	(5/84)[5]	
						Leuk	5-14	X	3709	Knox	0	(0/84)[5]	
Knox et al., 1992	England, Wales and Scotland, UK	1966-1983	3	Postcodes	s: 0.5, 1, 1.5, 2, 5, 20, 100 km t: 0.5, 1, 2, 4, 12 months	Leuk+Lymph	0-14	X	8888	Knox	0.143	(6/42)	Leuk&Lymph, 0-14y, 0.5km, 2mths; LL&unsp.Leuk, 0-14y, 0.5km, 0.5mth, Leuk&Lymph, 4-7y, 1.5km, 1mth
						LL*	0-14	X	NA	Knox	0.071	(3/42)	
						Leuk+Lymph	0-3	X	NA	Knox	0	(0/42)	
						Leuk+Lymph	4-7	X	NA	Knox	0.095	(4/42)	
						Leuk+Lymph	8-13	X	NA	Knox	0	(0/42)	
Schneider et al., 1993	New Jersey, USA	1979-1985	2	Areas	s: same minor civil division (town) t: 7, 14 months	Leuk	0-14	X	5500[6]	E-M-M	0.5	(1/2)	Leuk, 0-14y, 7mths

Table S1 continued

Author, Year	Study area	Period	Quality	Resolution	Critical lags	Cancer type	Age	Time	Cases	Method	Results		
											Rate of sig. Tests[a]		Significant test[b]
Gilman et al., 1995	England, Wales and Scotland, UK	1953-1980	3	Postcodes	s: 1, 2, 3, 4, 5, 10, 20 km<br>t: 0, 1, ..., 6, 9, 12, 18, 24, 48 months	Leuk+Lymph	0-15	B	1435	Knox	0.022	(1/45)[7]	B: Leuk+Lymph, 0-15y, 53-64, central Britain, 2km, 1mth, Leuk+Lymph, 0-15y, 53-64, outside central Britain, 1km, 3mths<br>X: Leuk+Lymph, 0-15y, central Britain, 2km, 2mths, Leuk+Lymph, 0-15y, outside central Britain, 4km, same mth
						Leuk+Lymph	0-15	B	2478	Knox	0.044	(2/45)[8]	
						Leuk+Lymph	0-15	X	3862	Knox	0.067	(3/45)[7]	
						Leuk+Lymph	0-15	X	6511	Knox	0.178	(8/45)[8]	
Petridou et al., 1996	Greece	1980-1989	2	Geocodes	s: Knox: 5 km; Global: 0.5-7.5 km<br>t: Knox: 1 year; Global: 1-15 months	Leuk	0-14	X	872	Knox	0.667	(2/3)	Knox: Leuk, 0-14y, 5km, 1y; Leuk, 0-4y, 5km, 1y; ALL, 0-14y, 5km, 1y; Global: Leuk, 0-14y; Leuk, 0-4y
						Leuk	0-4	X	446	Knox	1	(3/3)	
						Leuk	5-9	X	278	Knox	0	(0/3)	
						Leuk	10-14	X	148	Knox	0	(0/3)	
						ALL	0-14	X	739	Knox	0.667	(2/3)	
						ALL	0-4	X	392	Knox	0	(0/3)	
						ALL	5-9	X	239	Knox	0	(0/3)	
						ALL	10-14	X	108	Knox	0	(0/3)	
						Leuk	0-14	X	872	Bhopal et al. 1992	1	(1/1)	
						Leuk	0-4	X	446	Bhopal et al. 1992	1	(1/1)	
Alexander et al., 1998	13 European Countries and Queensland, Australia (Euroclus)	1980-1989	1	Areas	s: same small census area<br>t: 1.5 years (ALL, 2-4 years; Lk, 0-1 year), 2 years (Lk, 5-14 years)			X	970	Knox	NA	(NA/NA)[9]	ALL, 2-4y; Leuk, 5-14y
Gustafsson et al., 1999	Sweden	1973-1989	2	Areas	s: same municipality<br>t: 1, 3, 6, 12, 24 months	AL***	0-15	B	645	Knox	0	(0/5)	B: ALL, 5-15y, same m., 1mth<br>X: n.s.
						AL***	0-4	B	NA	Knox	0	(0/5)	
						AL***	5-15	B	NA	Knox	1	(1/1)	
						AL***	0-15	X	645	Knox	0	(0/5)	
						AL***	0-4	X	NA	Knox	0	(0/5)	
						AL***	5-15	X	NA	Knox	0	(0/5)	
Gilman et al., 1999	England and Wales, UK (approx. 40%)	1984-1993	3	Postcodes	s: 1, 2, 3, 4, 5 km<br>t: 1, 2, ..., 6, 9, 12, 18 months	ALL	0-14	X	255	Knox	0.12	(6/50)[10]	ALL, 0-14y, 84-88, 1km, 6mths; ALL, 0-14y, 89-93, 4km, same mth
						ALL	0-14	X	290	Knox	0.02	(1/50)[11]	

Table S1 continued

Author, Year	Study area	Period	Quality	Resolution	Critical lags	Cancer type	Age	Time	Cases	Method	Results		
											Rate of sig. Tests[a]		Significant test[b]
Birch et al., 2000	Greater Manchester, Lancashire, Cheshire, Cumbria, Derbyshire, UK	1954-1985	3	Geocodes	s: Knox: 5 km, NN=b75/x68; K-f: 0.5-7.5 km, NN=71-85/63-77<br>t: 1 year	Leuk	0-14	B	798	Knox	0	(0/2)	B: n.s.<br>X: Knox: Leuk, 0-14y, 5km, 1y; Leuk, 0-4y, 5km, 1y; K-f: Leuk, 0-14y, km; Leuk, 0-14y, NN; Leuk, 0-4y, km; ALL, 0-14y, km; ALL, 0-14y, NN
						Leuk	0-14	B	798	K-f	0	(0/2)	
						Leuk	0-4	B	410	Knox	0	(0/2)	
						Leuk	0-4	B	410	K-f	0	(0/2)	
						Leuk	5-9	B	230	Knox	NA	(NA/NA)	
						Leuk	5-9	B	230	K-f	NA	(NA/NA)	
						Leuk	10-14	B	158	Knox	NA	(NA/NA)	
						Leuk	10-14	B	158	K-f	NA	(NA/NA)	
						ALL	0-14	B	647	Knox	0	(0/2)	
						ALL	0-14	B	647	K-f	0	(0/2)	
						ALL	0-4	B	359	Knox	0	(0/2)	
						ALL	0-4	B	359	K-f	0	(0/2)	
						ALL	5-9	B	187	Knox	NA	(NA/NA)	
						ALL	5-9	B	187	K-f	NA	(NA/NA)	
						ALL	10-14	B	101	Knox	NA	(NA/NA)	
						ALL	10-14	B	101	K-f	NA	(NA/NA)	
						ALL	18-54 mths	B	292	Knox	0	(0/2)	
						ALL	18-54 mths	B	292	K-f	0	(0/2)	
						ANLL	0-14	B	151	Knox	0	(0/NA)	
						ANLL	0-14	B	151	K-f	0	(0/NA)	
						Leuk	0-14	X	908	Knox	0.5	(1/2)	
						Leuk	0-14	X	908	K-f	1	(2/2)	
						Leuk	0-4	X	457	Knox	0.5	(1/2)	
						Leuk	0-4	X	457	K-f	0.5	(1/2)	
						Leuk	5-9	X	268	Knox	NA	(NA/NA)	
						Leuk	5-9	X	268	K-f	NA	(NA/NA)	
						Leuk	10-14	X	183	Knox	NA	(NA/NA)	
						Leuk	10-14	X	183	K-f	NA	(NA/NA)	
						ALL	0-14	X	739	Knox	0	(0/2)	
						ALL	0-14	X	739	K-f	1	(2/2)	
						ALL	0-4	X	399	Knox	0	(0/2)	
						ALL	0-4	X	399	K-f	0	(0/2)	
						ALL	5-9	X	216	Knox	NA	(NA/NA)	
						ALL	5-9	X	216	K-f	NA	(NA/NA)	
						ALL	10-14	X	124	Knox	NA	(NA/NA)	
						ALL	10-14	X	124	K-f	NA	(NA/NA)	

Table S1 continued

Author, Year	Study area	Period	Quality	Resolution	Critical lags	Cancer type	Age	Time	Cases	Method	Results: Rate of sig. Tests[a]		Results: Significant test[b]
						ALL	18-54 mths	X	326	Knox	0	(0/2)	
						ALL	18-54 mths	X	326	K-f	0	(0/2)	
						ANLL	0-14	X	169	Knox	0	(0/NA)	
						ANLL	0-14	X	169	K-f	0	(0/NA)	
Gustafsson et al., 2000	Sweden	1973-1996	2	Areas	s: same municipality<br>t: 1, 3, 6, 12, 24 months	ALL	0-14	B	1020	Knox	0	(0/5)	B: ALL, 4-14y, same m., 1mth; ALL, 5-14y, same m., 1mth; ALL+unsp.Lk, 4-14, same m., 1mth<br>X: n.s.
						ALL	0-4	B	661	Knox	0	(0/5)	
						ALL	0-1	B	NA	Knox	0	(0/5)	
						ALL	1-5	B	NA	Knox	0	(0/5)	
						ALL	2-4	B	NA	Knox	0	(0/5)	
						ALL	2-6	B	NA	Knox	0	(0/5)	
						ALL	4-14	B	503	Knox	1	(1/1)	
						ALL	5-14	B	359	Knox	0.2	(1/5)	
						AL***	0-14	B	1257	Knox	0	(0/5)	
						AL***	0-4	B	NA	Knox	0	(0/5)	
						AL***	0-1	B	NA	Knox	0	(0/5)	
						AL***	1-5	B	NA	Knox	0	(0/5)	
						AL***	2-4	B	NA	Knox	0	(0/5)	
						AL***	2-6	B	NA	Knox	0	(0/5)	
						AL***	4-14	B	NA	Knox	0.2	(1/5)	
						AL***	5-14	B	NA	Knox	0	(0/5)	
						ALL	0-14	X	NA	Knox	0	(0/5)	
						ALL	0-4	X	NA	Knox	0	(0/5)	
						ALL	0-1	X	NA	Knox	0	(0/5)	
						ALL	1-5	X	NA	Knox	0	(0/5)	
						ALL	2-4	X	NA	Knox	0	(0/5)	
						ALL	2-6	X	NA	Knox	0	(0/5)	
						ALL	4-14	X	NA	Knox	0	(0/5)	
						ALL	5-14	X	NA	Knox	0	(0/5)	
						AL***	0-14	X	NA	Knox	0	(0/5)	
						AL***	0-4	X	NA	Knox	0	(0/5)	
						AL***	0-1	X	NA	Knox	0	(0/5)	
						AL***	1-5	X	NA	Knox	0	(0/5)	
						AL***	2-4	X	NA	Knox	0	(0/5)	
						AL***	2-6	X	NA	Knox	0	(0/5)	
						AL***	4-14	X	NA	Knox	0	(0/5)	
						AL***	5-14	X	NA	Knox	0	(0/5)	

Table S1 continued

Author, Year	Study area	Period	Quality	Resolution	Critical lags	Cancer type	Age	Time	Cases	Method	Results		
											Rate of sig. Tests[a]		Significant test[b]
McNally et al., 2002	North West England, UK	1980-2001	3	Postcodes	s: Knox: 5 km, NN=b40/x41; K-f: 0.5-7.5 km, NN=33-47/34-48 t: 1 year	ALL	0-14	B	512	Knox	0	(0/2)	B: K-f: Precursor B-cell ALL, 18-54mths, NN, 1y X: n.s.
						ALL	0-14	B	512	K-f	0	(0/2)	
						ALL	0-14	X	512	Knox	0	(0/2)	
						ALL	0-14	X	512	K-f	0	(0/2)	
Akhtar et al., 2005	Karachi, Pakistan	1995-2002	1	Geocodes	s: 1, 1.5, 2, ..., 7 km t: 1, 2, 3, 4, 5, 6 months	Leuk	0-14	X	142	Knox	0.013	(1/78)	Leuk, 0-14y, 6km, 3mths
McNally et al., 2006	Great Britain	1969-1993	3	Postcodes	s: K-f: NN=19-33, Knox: not specified t: K-f: 0.1, 0.2, ..., 1.5 years; Knox: not specified	Leuk	0-14	X	10727	K-f	1	(1/1)	K-f: Leuk, 0-14y; ALL, 0-14y; ALL, 1-4y
						Leuk	1-4	X	5092	K-f	0	(0/1)	
						Leuk	5-14	X	5087	K-f	0	(0/1)	
						ALL	0-14	X	8439	K-f	1	(1/1)	
						ALL	1-4	X	4343	K-f	1	(1/1)	
						ALL	5-14	X	3810	K-f	0	(0/1)	
						ANLL	0-14	X	1736	K-f	0	(0/1)	
Bellec et al., 2006	France	1990-2000	2	Areas	s: Knox: 0, 5, 10, 15, 20, 30, 50 km; K: 10% of population t: Knox: 1, 3, 6, 9, 12 months; K: 50% of time	AL	0-14	X	4873	Knox	0	(0/35)	Knox: AL, 0-4y, 30km, 3mths; AL, 10-14y, 0km, 6mths; K: n.s.
						AL	0-4	X	2458	Knox	0.029	(1/35)	
						AL	5-9	X	1427	Knox	0	(0/35)	
						AL	10-14	X	988	Knox	0.029	(1/35)	
						AL	0-14	X	4873	Scan	0	(0/1)	
						AL	0-4	X	2458	Scan	0	(0/1)	
						AL	5-9	X	1427	Scan	0	(0/1)	
						AL	10-14	X	988	Scan	0	(0/1)	
COMARE, 2006	England, Scotland and Wales, UK	1969-1993	3	Postcodes	s: 0.5, 1, 1.5, ..., 7.5 km t: 0.1, 0.2, ..., 1.5 years	Leuk	0-14	X	10737	K-f	1	(1/1)	Leuk, 0-14y; ALL, 0-14y; ALL, 1-4y
						Leuk	1-4	X	5094	K-f	0	(0/1)	
						Leuk	5-14	X	5092	K-f	0	(0/1)	
						ALL	0-14	X	8687	K-f	1	(1/1)	
						ALL	1-4	X	4441	K-f	1	(1/1)	
						ALL	5-14	X	3906	K-f	0	(0/1)	
Kearney, 2008	Florida, USA	1990-1999	3	Areas	s: Census Tracts t: 1-10 years	Leuk	0-19	X	1125	Scan	1	(1/1)	Leuk, 0-19y
McNally et al., 2009	Great Britain	1969-1993	3	Postcodes	s: K-f: NN=18-32, Knox: NN=25 t: K-f: 0.1, 0.2, ..., 1.5 years; Knox: 1 year	Leuk	0-14	B	9763	K-f	0	(0/1)	n.s.
						Leuk	1-4	B	4850	K-f	0	(0/1)	
						Leuk	5-14	B	4365	K-f	0	(0/1)	
						ALL	0-14	B	7759	K-f	0	(0/1)	
						ALL	1-4	B	4140	K-f	0	(0/1)	
						ALL	5-14	B	3335	K-f	0	(0/1)	
						ANLL	0-14	B	1528	K-f	0	(0/1)	
Schmiedel et al., 2010	Germany	1987-2007	2	Areas	s: municipalities t: individual years	Leuk	0-14	X	11946	Scan	0	(0/1)	n.s.
						Leuk	0-4	X	6103	Scan	0	(0/1)	
						Leuk	5-9	X	3407	Scan	0	(0/1)	

Table S1 continued

Author, Year	Study area	Period	Quality	Resolution	Critical lags	Cancer type	Age	Time	Cases	Method	Results		
											Rate of sig. Tests[a]		Significant test[b]
						Leuk	10-14	X	2436	Scan	0	(0/1)	
Amin et al., 2010	Florida, USA	2000-2007	3	Areas	s: not specified<br>t: individual years (not specified)	Leuk	0-19	X	1254	Scan	1	(1/1)	Leuk, 0-19y, 00-02
Demoury et al., 2012	France	1990-2006	2	Areas	s: 100 living zones<br>t: individual years	AL	0-14	X	7675	Scan	0	(0/3)	n.s.
						AML	0-14	X	1276	Scan	0	(0/3)	
						ALL	0-14	X	6269	Scan	0	(0/3)	
						ALL	1-4	X	3013	Scan	0	(0/3)	
Ortega-Garcia et al., 2015	Murcia, Spain	1998-2013	2	Areas	s: census tracts<br>t: months	Leuk	0-14	X	39	Scan	0	(0/1)	n.s.
Kreis et al., 2016	Switzerland	1985-2010	4	Geocodes	s: 0.5, 1, 2, 5, 10 km<br>t: 6, 12, 18, 24 months	Leuk	0-15	B	1052	Knox	1	(1/1)	B: Leuk, 0-15y, 1km, 2y; ALL, 0-15y, 1km, 2y<br>X: n.s.
						Leuk	0-4	B	631	Knox	0	(0/1)	
						Leuk	5-15	B	421	Knox	0	(0/1)	
						Leuk	0-15	X	1485	Knox	0	(0/1)	
						Leuk	0-4	X	753	Knox	0	(0/1)	
						Leuk	5-15	X	732	Knox	0	(0/1)	
						ALL	0-15	B	852	Knox	0	(0/1)	
						ALL	0-4	B	519	Knox	0	(0/1)	
						ALL	5-15	B	333	Knox	0	(0/1)	
						ALL	0-15	X	1185	Knox	0	(0/1)	
						ALL	0-4	X	628	Knox	0	(0/1)	
						ALL	5-15	X	557	Knox	0	(0/1)	

[a]Proportion of significant space-time clustering tests per analysis group (number of clustering tests signficant at 5% alpha-level/total number of clustering tests performed).

[b]Characteristics of (most) significant space-time clustering test, specifying (if applicable): time frame of birth B: or diagnosis X: clustering test: diagnostic group, age range, spatial lag, temporal lag, sub-area, sub-period. Results are not adjusted for multiple testing.

Critical lags: *s* spatial lag, *t* temporal lag, *NN* nearest neighbors, *M* Mantel test, *K-f* K-functions, *K* Kulldorff's scan statistic; **Cancer type:** *Leuk* Leukaemia, *AL* acute leukaemia, *AL** AL and chronic granulocytic leukaemia, *AL*** acute granulocytic and monocytic leukaemia, *ALL* acute lymphoblastic leukemia, *AL**** ALL and acute unspecified leukaemia, *LL* Lymphoid leukemia, *LL** lymphocytic and unspecified leukaemia, *ML* myeloid leukaemia, *AML* acute myeloid leukaemia; **Time:** *B* date of birth, *X* date of diagnosis; **Method:** *E-M-M* Ederer-Myers-Mantel, *K-f* K-functions, *K statistic* combinatorial test invented by the authors, *Scan* Kulldorff's scan statistic; **Results:** *NA* not applicable, *n.s.* not significant, *M* Mantel test, *K-f* K-functions, *K* Kulldorff's scan statistic, *m* mile(s), *d* days, *mth(s)* month(s), *y* year(s).

[1]Not counting Knox analyses used for descriptive purposes rather than significance testing purposes

[2]Two tests performed for two sub-periods 1956-67 and 1957-68

[3]1952-1959

[4]1961-1964

[5]Summary statistic for whole period used

[6]All diagnostic groups (not only leukaemias)

[7]Inside central Britain

[8]Outside central Britain

[9]Excluded from quantiative synthesis as space-time clustering analyses were performed on census areas that were pre-identified as containing case excesses

[10]1984-1988

[11]1989-1993
