# Supplementary material for: Space–time clustering of childhood cancers: a systematic review and pooled analysis
Source: Eur J Epidemiol. 2018 Nov 16;34(1):9–21. doi: 10.1007/s10654-018-0456-y (PMC6326085; doi:10.1007/s10654-018-0456-y)
Supplement: Supplementary file 4 — Supplementary material 4 (PDF 135 kb) [file 10654_2018_456_MOESM4_ESM.pdf]

Table S2 Space-time clustering studies of lymphoma

Author, Year	Study Area	Period	Quality	Resolution	Critical lags	Type of cancer	Age	Time	Cases	Method	Results		
											Rate of sig. Tests[a]		Significant test[b]
Pinkel et al., 1963	Buffalo Area, NY, USA	1943-1956	2	Postcodes	s: 1/8, 2/8, ..., 1 mile<br>t: not specified	HL	0-15	X	206	Average ridit	NA	(NA/NA)[1]	
Ederer et al., 1965	State of Connecticut, USA	1945-1959	2	Areas	s: same town<br>t: 1, 2 years	Lymphoma	0-14	X	448	E-M-M	0	(0/2)	n.s.
Pike et al., 1967	West Nile District, Uganda	1961-1965	1	Geocodes	s: 2, 5, 10, 20, 40 km<br>t: 30, 60, 90, 120, 180, 360 days	BL	0-12	X	36	Knox	0.633	(19/30)	BL, 0-12y, 40km, 180d
						BL	0-12	X	36	David & Barton 1966	NA	(NA/NA)[2]	
Glass et al., 1968	Los Angeles County, USA	1960-1964	1	Geocodes	s: same census region<br>t: 1 year	Lymphoma	0-14	X	645	E-M-M	NA	(NA/NA)[1]	
Williams et al., 1969	West Nile District, Uganda	1966-1967	1	Geocodes	s: 1, 2, 3, 4, 5, 6, 7, 8, 9, 10 km<br>t: 2 years	BL	0-10	X	29	Knox	0.6	(6/10)	BL, 0-10y, 9km, 2y
Fraumeni et al., 1969	United States of America	1960-1964	0	Areas	s: same region; same division; same state<br>t: 1 year	HL	0-14	X	359	E-M-M	0	(0/3)[3]	n.s.
Brubaker et al., 1973	North Mara District, Tanzania	1964-1970	2	Geocodes	s: 1, 2, 4, 8, 16, 32 km<br>t: 0.25, 0.5, 1, 2, 3, 4, 6 months	BL	0-14	X	36	Knox	0	(0/42)	n.s.
Morrow et al., 1976	Mengo District, Uganda	1959-1968	2	Geocodes	s: 2.5, 5, 10, 20, 40 km<br>t: Knox: 30, 60, 90, 12, 180, 360 days; Barton: 5, 10, 15, 20, 25, 45, 60, 75, 120, 180 days	BL	0-19[4]	X	113	Knox	0	(0/30)	n.s.
						BL	0-19[4]	X	113	Barton 1965	0	(0/50)	
Morrow et al., 1977	Lango and Acholi districts, Uganda	1963-1968	2	Geocodes	s: 2.5, 5, 10, 20, 40 km<br>t: 30, 60, 90, 120, 180, 360 days	BL	0-14+	X	85	Knox	0	(0/42)	n.s.
Williams et al., 1978	West Nile District, Uganda	1961-1975	2	Geocodes	s: 2.5, 5, 10, 20, 40 km<br>t: 1, 2, 3, 4, 6, 12 months	BL	0-15+	X	35	Knox	0.633	(19/30)[5]	BL, 0-15y, 40km, 4mths, 61-65; BL, 0-15y, 10km, 12mths, 66-70, BL, 0-15y, 10km, 6mths, 71-75; BL, 0-15y, 10km, 6mths, 72-73
						BL	0-15+	X	72	Knox	0.067	(2/30)[6]	
						BL	0-15+	X	81	Knox	0.1	(3/30)[7]	
Biggar et al., 1979	Ghana	1970-1975	0	Areas	s: 40km<br>t: 3, 6, 9, 12, 18, 24, 36 months	BL, Non-BL	NA	X	339	Knox (modified)	0.286	(2/7)	BL, na, 40km, 3mths

Table S2 continued

Author, Year	Study Area	Period	Quality	Resolution	Critical lags	Type of cancer	Age	Time	Cases	Method	Results		
											Rate of sig. Tests[a]		Significant test[b]
Siemiatycki et al., 1980	North Mara, Tanzania; West Nile, Uganda	1961-1977	1	Geocodes	s: Knox: 5, 20 km; M: 24 combinations of time and space functions<br>t: Knox: 4, 12 months; M: 24 combinations of time and space functions	BL	0-15	X	278	Knox, Mantel	0	(0/24)[8]	BL, 0-15y, 20km, 12mths, West Nile, 61-65; North Mara n.s.
						BL	0-15	X	278	Knox, Mantel	0	(0/24)[9]	
						BL	0-15	X	278	Knox, Mantel	0.458	(11/24)[10]	
						BL	0-15	X	278	Knox, Mantel	0	(0/24)[11]	
						BL	0-15	X	278	Knox, Mantel	0	(0/24)[12]	
Mangoud et al., 1985	Greater Manchester, UK	1962-1976	2	Postcodes	s: 0.5, 1, 2, 4, 8, 16 km<br>t: 1, 2, 4, 8, 12, 24, 36, 48, 60, 72, 84 months	HL	0-14	X	39	Knox	0.197	(13/66)	HL, 0-14y, 1km, 1mth
Gilman et al., 1991	England, Scotland and Wales, UK	1966-1983	3	Postcodes	s: 1, 2, 3, 4, 5, 10, 20 km<br>t: 0, 1, ..., 9, 12, 18, 24, 48 months	NHL	0-14	X	1251	Knox	0.071	(6/84)[13]	NHL, 0-14, 3km, 0mths; NH+unsp.Lymph, 0-14y, 3km, 0mths; NH+unsp.Lymph, 0-4y, 2km, 0mths; NH+unsp.Lymph, 5-14y, 5km, 24mths
						NH and unspecified Lymphoma	0-14	X	1363	Knox	0.06	(5/84)[13]	
						NH and unspecified Lymphoma	0-4	X	332	Knox	0.024	(2/84)[13]	
						NH and unspecified Lymphoma	5-14	X	1031	Knox	0.048	(4/84)[13]	
Knox et al., 1992	England, Wales and Scotland, UK	1966-1983	3	Postcodes	s: 0.5, 1, 1.5, 2, 5, 20, 100 km<br>t: 0.5, 1, 2, 4, 12 months	Leuk+Lymph	0-14	X	8888	Knox	0.143	(6/42)	Leuk&Lymph, 0-14y, 0.5km, 2mths; Leuk&Lymph, 4-7y, 1.5km, 1mth
						Leuk+Lymph	0-3	X	NA	Knox	0	(0/42)	
						Leuk+Lymph	4-7	X	NA	Knox	0.095	(4/42)	
						Leuk+Lymph	8-13	X	NA	Knox	0	(0/42)	
Van Den Bosch et al., 1993	Malawi	1987-1989	1	Geocodes	s: 2.5, 5, 10, 15, 20 km<br>t: 0.33, 1, 2, 3, 4, 6 months	BL	0-22	X	146	Knox	0.033	(1/30)	BL, 0-22y, 2.5km, 2mths; BL, 8-22y, 2.5km, 2mths; BL, 8-22y, 5km, 3mths
						BL	8-22	X	72	Knox	0.067	(2/30)	
Schneider et al., 1993	New Jersey, USA	1979-1985	2	Areas	s: same minor civil division (town)<br>t: 7, 14 months	HL	0-14	X	5500[14]	E-M-M	0.5	(1/2)	HL, 0-14y, 7mths; NHL, 0-14y, 7mths
						NHL	0-14	X	5500[14]	E-M-M	0.5	(1/2)	

Table S2 continued

Author, Year	Study Area	Period	Quality	Resolution	Critical lags	Type of cancer	Age	Time	Cases	Method	Results	
											Rate of sig. Tests[a]	Significant test[b]
Gilman et al., 1995	England, Wales and Scotland, UK	1953-1980	3	Postcodes	s: 1, 2, 3, 4, 5, 10, 20 km<br>t: 0, 1, ..., 6, 9, 12, 18, 24, 48 months	Leuk+Lymph	0-15	B	1435	Knox	0.022 (1/45)[15]	B: Leuk+Lymph, 0-15y, 53-64, central Britain, 2km, 1mth, Leuk+Lymph, 0-15y, 53-64, outside central Britain, 1km, 3mths<br>X: Leuk+Lymph, 0-15y, central Britain, 2km, 2mths, Leuk+Lymph, 0-15y, outside central Britain, 4km, same mth
						Leuk+Lymph	0-15	B	2478	Knox	0.044 (2/45)[16]	
						Leuk+Lymph	0-15	X	3862	Knox	0.067 (3/45)[15]	
						Leuk+Lymph	0-15	X	6511	Knox	0.178 (8/45)[16]	
Gilman et al., 1999	England and Wales (approx. 40%)	1984-1993	3	Geocodes	s: 1, 2, 3, 4, 5 km<br>t: 0, 1, ..., 6, 9, 12, 18 months	HL	0-14	X	47	Knox	0 (0/1)[17]	HL, 0-14y, 2km, same mth, 89-93
						HL	0-14	X	34	Knox	0 (0/1)[18]	
Gustafsson et al., 2000	Sweden	1973-1996	2	Areas	s: same municipality<br>t: 1, 3, 6, 12, 24 months	ALL + NHL	0-14	B	1313	Knox	0 (0/5)	B: n.s.<br>X: n.s.
						ALL + NHL	0-4	B	791	Knox	0 (0/5)	
						ALL + NHL	0-1	B	NA	Knox	0 (0/5)	
						ALL + NHL	1-5	B	NA	Knox	0 (0/5)	
						ALL + NHL	2-4	B	NA	Knox	0 (0/5)	
						ALL + NHL	2-6	B	NA	Knox	0 (0/5)	
						ALL + NHL	4-14	B	688	Knox	0 (0/5)	
						ALL + NHL	5-14	B	522	Knox	0 (0/5)	
						ALL + NHL	0-14	X	NA	Knox	0 (0/5)	
						ALL + NHL	0-4	X	NA	Knox	0 (0/5)	
						ALL + NHL	0-1	X	NA	Knox	0 (0/5)	
						ALL + NHL	1-5	X	NA	Knox	0 (0/5)	
						ALL + NHL	2-4	X	NA	Knox	0 (0/5)	
						ALL + NHL	2-6	X	NA	Knox	0 (0/5)	
						ALL + NHL	4-14	X	NA	Knox	0 (0/5)	
						ALL + NHL	5-14	X	NA	Knox	0 (0/5)	

Table S2 continued

Author, Year	Study Area	Period	Quality	Resolution	Critical lags	Type of cancer	Age	Time	Cases	Method	Results		
											Rate of sig. Tests[a]		Significant test[b]
McNally et al., 2004	North West England, UK	1954-2001	3	Postcodes	s: Knox: 5 km, NN=b11/x11; K-f: 0.5-7.5 km, NN=4-18/4-18<br>t: Knox: 1 year; K-f: 1-15 months	Lymphoma	0-14	B	414	K-f	0	(0/2)	B: n.s.<br>X: n.s.
						HL	0-14	B	188	K-f	0	(0/2)	
						NHL	0-14	B	211	K-f	0	(0/2)	
						HL+NHL	0-14	B	399	K-f	0	(0/2)	
						Lymphoma	0-14	B	414	Knox	0	(0/2)	
						HL	0-14	B	188	Knox	0	(0/2)	
						NHL	0-14	B	211	Knox	0	(0/2)	
						HL+NHL	0-14	B	399	Knox	0	(0/2)	
						Lymphoma	0-14	X	414	K-f	0	(0/2)	
						HL	0-14	X	188	K-f	0	(0/2)	
						NHL	0-14	X	211	K-f	0	(0/2)	
						HL+NHL	0-14	X	399	K-f	0	(0/2)	
						Lymphoma	0-14	X	414	Knox	0	(0/2)	
						HL	0-14	X	188	Knox	0	(0/2)	
						NHL	0-14	X	211	Knox	0	(0/2)	
						HL+NHL	0-14	X	399	Knox	0	(0/2)	
McNally et al., 2006	Great Britain	1969-1993	3	Postcodes	s: K-f: NN=19-33, Knox: not specified<br>t: K-f: 0.1, 0.2, ..., 1.5 years; Knox: not specified	Lymphoma	0-14	X	3306	K-f	0	(0/1)	n.s.
						HL	0-14	X	1364	K-f	0	(0/1)	
						HL	0-9	X	487	K-f	0	(0/1)	
						NHL	0-14	X	1678	K-f	0	(0/1)	
						NHL	0-9	X	1027	K-f	0	(0/1)	
COMARE, 2006	England, Scotland and Wales, UK	1969-1993	3	Postcodes	s: 0.5, 1, 1.5, ..., 7.5 km<br>t: 0.1, 0.2, ..., 1.5 years	HL	0-14	X	877	K-f	0	(0/1)	n.s.
						HL	0-9	X	487	K-f	0	(0/1)	
						NHL	0-14	X	1678	K-f	0	(0/1)	
						NHL	0-9	X	1027	K-f	0	(0/1)	
Kearney, 2008	Florida, USA	1990-1999	3	Areas	s: Census Tracts<br>t: 1-10 years	Lymphoma (not including HL)	0-19	X	126	Scan	1	(1/1)	Lymphoma, 0-19y
McNally et al., 2009	Great Britain	1969-1993	3	Postcodes	s: K-f: NN=18-32, Knox: NN=25<br>t: K-f: 0.1, 0.2, ..., 1.5 years; Knox: 1 year	Lymphoma	0-14	B	2939	K-f	0	(0/1)	HL, 0-14y
						HL	0-14	B	1226	K-f	1	(1/1)	
						HL	0-9	B	444	K-f	0	(0/1)	
						NHL	0-14	B	1485	K-f	0	(0/1)	
						NHL	0-9	B	937	K-f	0	(0/1)	
Amin et al., 2010	Florida, USA	2000-2007	3	Areas	s: not specified<br>t: individual years (not specified)	Lymphoma	0-19	X	252	Scan	0	(0/1)	n.s.
Ortega-Garcia et al., 2015	Murcia, Spain	1998-2013	2	Areas	s: census tracts<br>t: months	Lymphoma	0-14	X	17	Scan	1	(1/1)	Lymph, 0-14y, 2 tracts, 07/11-06/13

Table S2 continued

Author, Year	Study Area	Period	Quality	Resolution	Critical lags	Type of cancer	Age	Time	Cases	Method	Results	
											Rate of sig. Tests[a]	Significant test[b]
Kreis et al., 2016	Switzerland	1985-2010	4	Geocodes	s: 0.5, 1, 2, 5, 10 km	Lymphoma	0-15	B	353	Knox	0 (0/1)	B: n.s.
					t: 6, 12, 18, 24 months	Lymphoma	0-15	X	635	Knox	0 (0/1)	X: n.s.

[a]Proportion of significant space-time clustering tests per analysis group (number of clustering tests signficant at 5% alpha-level/total number of clustering tests performed).

[b]Characteristics of (most) significant space-time clustering test, specifying (if applicable): time frame of birth B: or diagnosis X: clustering test: diagnostic group, age range, spatial lag, temporal lag, sub-area, sub-period. Results are not adjusted for multiple testing.

Critical lags: *s* spatial lag, *t* temporal lag, *NN* nearest neighbors; **Type of Cancer:** *HL* Hodgkin lymphoma, *NHL* non-Hodgkin lymphoma, *BL* Burkitt lymphoma; **Time**: *B* date of birth, *X* date of diagnosis; **Method:** *E-M-M* Ederer-Myers-Mantel, *K-f* K-functions, *Scan* Kulldorff's Scan Statistic; **Results:** *NA* not available, *n.s.* not significant

[1]Excluded as lymphoma were not analyzed as separate diagnostic group

[2]Excluded as number of tests performed is unclear

[3]E-M-M summed for all states, division and regions

[4]Separate analysis of children under 16 years

[5]1961-1965; study group identical with Pike et al. 1967

[6]1966-1970

[7]1971-1975

[8]North Mara, 1964-1970

[9]North Mara, 1971-1977

[10]West Nile, 1961-1965

[11]West Nile, 1966-1970

[12]West Nile, 1971-1976

[13]Summary statistic for whole period used

[14]All diagnostic groups (not only lymphomas)

[15]Inside central Britain

[16]Outside central Britain

[17]1984-1988, Bonferroni corrected

[18]1989-1993, Bonferroni corrected
