# Supplementary material for: Space–time clustering of childhood cancers: a systematic review and pooled analysis
Source: Eur J Epidemiol. 2018 Nov 16;34(1):9–21. doi: 10.1007/s10654-018-0456-y (PMC6326085; doi:10.1007/s10654-018-0456-y)
Supplement: Supplementary file 5 — Supplementary material 5 (PDF 117 kb) [file 10654_2018_456_MOESM5_ESM.pdf]

Table S3 Space-time clustering studies of childhood CNS tumours

Author, Year	Study area	Period	Quality	Resolution	Critical lags	Cancer type	Age	Time	Cases	Method	Results	
											Rate of sig. Tests[a]	Significant test[b]
Pinkel et al., 1963	Buffalo Area, NY, USA	1943-1956	2	Postcodes	s: 1/8, 2/8, ..., 1 mile<br>t: not specified	Brain and spinal cord tumors	0-15	X	NA	Average ridit	NA (NA/NA)[1]	
Glass et al., 1968	Los Angeles County, USA	1960-1964	1	Geocodes	s: same census region<br>t: 1 year	Brain tumors	0-14	X	NA	E-M-M	NA (NA/NA)[1]	
Morris, 1990	Worcestershire, Warwickshire, Staffordshire, Shropshire; UK	1953-1960	2	Geocodes	s: not specified<br>t: not specified	Cerebral tumours	0-9	X	99	Knox	NA (NA/NA)[1]	
Schneider et al., 1993	New Jersey, USA	1979-1985	2	Areas	s: same minor civil division (town)<br>t: 7, 14 months	Brain/CNS	0-14	X	5500[2]	E-M-M	0 (0/2)	n.s.
Hjalmars et al., 1999	Sweden	1973-1992	3	Areas	s: Knox: 0, 2, 5, 10, 20, 50, 100 km; K: 10% of population<br>t: 0, 3, 6, 12, 24 months	Astrocytoma	0-15	X	628	Knox	0 (0/1)[3]	n.s.
						Astrocytoma, Medulloblastoma and Ependymoma	0-15	X	938	Knox	0 (0/1)[3]	
						Astrocytoma	0-15	X	628	Scan	0 (0/1)	
						Astrocytoma, Medulloblastoma and Ependymoma	0-15	X	938	Scan	0 (0/1)	
McNally et al., 2002	North West England, UK	1954-1998	3	Postcodes	s: Knox: 5 km, NN=b31/x30; K-f: 0.5-7.5 km, NN=23-37<br>t: Knox: 1 year; K-f: 1-15 months	CNS	0-14	B	1045	K-f	NA (NA/NA)	B: significant but no results specified<br>X: Knox: CNS, 0-14y, NN; Astro, 0-14y; Astro, 5-14y, 5km, 1y; K-f: CNS, 0-14y, NN; Astro, 0-14y, NN
						CNS	0-14	X	1045	K-f	0.5 (1/2)	
						CNS	0-14	X	1045	Knox	0.5 (1/2)	
						Astrocytoma	0-14	X	422	K-f	0.5 (1/2)	
						Astrocytoma	0-14	X	422	Knox	1 (2/2)	
						Astrocytoma	0-4	X	118	K-f	0 (0/2)	
						Astrocytoma	0-4	X	118	Knox	0 (0/2)	
						Astrocytoma	5-14	X	304	K-f	0 (0/2)	
						Astrocytoma	5-14	X	304	Knox	0.5 (1/2)	
						Ependymoma	0-14	X	109	K-f	0 (0/2)	
						Ependymoma	0-14	X	109	Knox	0 (0/2)	
						Medulloblastoma; PNET	0-14	X	200	K-f	0 (0/2)	
						Medulloblastoma; PNET	0-14	X	200	Knox	0 (0/2)	

Table S3 continued

Author, Year	Study area	Period	Quality	Resolution	Critical lags	Cancer type	Age	Time	Cases	Method	Results: Rate of sig. Tests[a]	Results: Significant test[b]
Houben et al., 2005	North Brabant, Netherlands	1983-2001	3	Postcodes	s: Knox: 5 km, NN=30; K-f: 0.5-7.5 km, NN=23-37<br>t: Knox: 1 year; K-f: 0.1-1.5 years	Glioma	0-14	X	56	Knox	0 (0/2)[4]	n.s.
						Glioma	0-14	X	45	Knox	0 (0/2)[5]	
						Glioma	0-14	X	56	K-f	0 (0/2)[6]	
						Glioma	0-14	X	45	K-f	0 (0/2)[7]	
McNally et al., 2006	Great Britain	1969-1993	3	Postcodes	s: K-f: NN=19-33, Knox: not specified<br>t: K-f: 0.1, 0.2, ..., 1.5 years; Knox: not specified	CNS	0-14	X	7469	K-f	0 (0/1)	n.s.
						Astrocytoma	0-14	X	2824	K-f	0 (0/1)	
						PNET	0-14	X	1548	K-f	0 (0/1)	
COMARE, 2006	England, Scotland and Wales, UK	1969-1993	3	Postcodes	s: 0.5, 1, 1.5, ..., 7.5 km<br>t: 0.1, 0.2, ..., 1.5 years	CNS	0-14	X	7473	K-f	0 (0/1)	n.s.
						Astrocytoma	0-14	X	2826	K-f	0 (0/1)	
Kearney, 2008	Florida, USA	1990-1999	3	Areas	s: Census Tracts<br>t: 1-10 years	Brain/CNS	0-19	X	1884	Scan	1 (1/1)	Brain/CNS, 0-19y
McNally et al., 2009	Great Britain	1969-1993	3	Postcodes	s: K-f: NN=18-32, Knox: NN=25<br>t: K-f: 0.1, 0.2, ..., 1.5 years; Knox: 1 year	CNS	0-14	B	6863	K-f	1 (1/1)	CNS, 0-14y
						Astrocytoma	0-14	B	2614	K-f	0 (0/1)	
						PNET	0-14	B	1410	K-f	0 (0/1)	
Amin et al., 2010	Florida, USA	2000-2007	3	Areas	s: not specified<br>t: individual years (not specified)	CNS/Brain tumor	0-19	X	4591	Scan	1 (1/1)	CNS, 0-19y, 06-07
Ortega-Garcia et al., 2011	Murcia, Spain	1998-2009	2	Areas	s: BHA<br>t: months (exact unit unclear)	CNS	0-14	X	91	Scan	0 (0/1)	Astrocytoma, 0-14y, 28 BHA, 10/02-02/05
						Astrocytoma	0-14	X	49	Scan	1 (1/1)	
						Medulloblastoma	0-14	X	20	Scan	0 (0/1)	
McNally et al., 2012	Yorkshire, UK	1974-2006	3	Postcodes	s: Knox/K-f: 0.5-7.5 km, K-f: NN=b1-15/x5-19; K: 10% of area<br>t: Knox/K-f: 0.1-1.5 years; K: 10% of time	CNS	0-14	B	506	K-f	0 (0/2)	B: K: CNS, 0-14y, 1982<br>X: K-f: PNET, 0-14y; PNET, 0-14y, NN, 74-90; PNET, 0-14y, NN, 91-06; Knox: PNET, 0-14y, 0.5km, 0.9y; K: CNS, 0-14y, 1975
						CNS	0-14	X	693	K-f	0 (0/2)	
						Ependymoma	0-14	B	46	K-f	0 (0/2)	
						Ependymoma	0-14	X	59	K-f	0 (0/2)	
						Astrocytoma	0-14	B	214	K-f	0 (0/2)	
						Astrocytoma	0-14	X	293	K-f	0 (0/2)	
						PNET	0-14	B	120	K-f	0 (0/2)	
						PNET	0-14	X	165	K-f	1 (2/2)	
						PNET	0-14	X	165	Knox	0.147 (33/225)	
						Other gliomas	0-14	B	58	K-f	0 (0/2)	
						Other gliomas	0-14	X	79	K-f	0 (0/2)	
						CNS	0-14	B	506	Scan	1 (1/1)	
						CNS	0-14	X	693	Scan	1 (1/1)	
						Ependymoma	0-14	B	46	Scan	0 (0/1)	
						Ependymoma	0-14	X	59	Scan	0 (0/1)	
						Astrocytoma	0-14	B	214	Scan	0 (0/1)	

Table S3 continued

Author, Year	Study area	Period	Quality	Resolution	Critical lags	Cancer type	Age	Time	Cases	Method	Results		
											Rate of sig. Tests[a]		Significant test[b]
						Astrocytoma	0-14	X	293	Scan	0	(0/1)	
						PNET	0-14	B	120	Scan	0	(0/1)	
						PNET	0-14	X	165	Scan	0	(0/1)	
						Other gliomas	0-14	B	58	Scan	0	(0/1)	
						Other gliomas	0-14	X	79	Scan	0	(0/1)	
Ortega-Garcia et al., 2015	Murcia, Spain	1998-2013	2	Areas	s: census tracts<br>t: months	CNS	0-14	X	38	Scan	0	(0/1)	n.s.
Kreis et al., 2016	Switzerland	1985-2010	4	Geocodes	s: 0.5, 1, 2, 5, 10 km<br>t: 6, 12, 18, 24 months	CNS	0-15	B	689	Knox	0	(0/1)	B: n.s.
						CNS	0-15	X	996	Knox	0	(0/1)	X: n.s.

[a]Proportion of significant space-time clustering tests per analysis group (number of clustering tests signficant at 5% alpha-level/total number of clustering tests performed).

[b]Characteristics of (most) significant space-time clustering test, specifying (if applicable): time frame of birth B: or diagnosis X: clustering test: diagnostic group, age range, spatial lag, temporal lag, sub-area, sub-period. Results are not adjusted for multiple testing.

Critical lags: *s* spatial lag, *t* temporal lag, *NN* nearest neighbors, *M* Mantel test, K-f K-functions, *K* Kulldorff's scan statistic, *BHA* Basic Health Area; **Cancer type:** *CNS* central nervous system tumour, *PNET* primitive neuroectodermal tumours, *Astro* Astrocytoma ; **Time:** *B* date of birth, *X* date of diagnosis; **Method:** *E-M-M* Ederer-Myers-Mantel, *K-f* K-functions, *Scan* Kulldorff's scan statistic; **Results:** *NA* not applicable, *n.s.* not significant, *M* Mantel test, *K-f* K-functions, *K* Kulldorff's scan statistic, *m* mile(s), *d* days, *mth(s)* month(s), *y* year(s).

[1]Excluded as CNS tumours were not analyzed as separate diagnostic group

[2]All diagnostic groups (not only CNS tumours)

[3]Knox adjusted for population shifts; combined p-value

[4]East, 1983-2001; [5]West, 1989-2001; [6]East, 1983-2001; [7]West, 1989-2001
