# Supplementary material for: Space–time clustering of childhood cancers: a systematic review and pooled analysis
Source: Eur J Epidemiol. 2018 Nov 16;34(1):9–21. doi: 10.1007/s10654-018-0456-y (PMC6326085; doi:10.1007/s10654-018-0456-y)
Supplement: Supplementary file 6 — Supplementary material 6 (PDF 127 kb) [file 10654_2018_456_MOESM6_ESM.pdf]

Table S4 Space-time clustering studies of other solid tumours

Author, Year	Study area	Period	Quality	Resolution	Critical lags	Cancer type	Age	Time	Cases	Method	Results		
											Rate of sig. Tests[a]		Significant test[b]
Pinkel et al., 1963	Buffalo Area, NY, USA	1943-1956	2	Postcodes	s: 1/8, 2/8, ..., 1 mile<br>t: not specified	Neuroblastoma; Wilms tumor; STS	0-15	X	NA	Average ridit	NA	(NA/NA)[1]	
Glass et al., 1968	Los Angeles County, USA	1960-1964	1	Geocodes	s: same census region<br>t: 1 year	Wilms tumor; Neuroblastoma; other	0-14	X	NA	E-M-M	NA	(NA/NA)[1]	
Miller et al., 1968	United States of America	1960-1964	0	Areas	s: same state; same standard division<br>t: 1 year	Neuroblastoma	0-14	X	1362	E-M-M	0	(0/1)[2]	Neuroblastoma, 0-14y, 3 standard divisions; Neuroblastoma, 0-
						Neuroblastoma	0-14	X	1362	E-M-M	1	(1/1)[3]	
Li et al., 1969	United States of America	1960-1964	0	Areas	s: same division<br>t: 1 year	Rhabdomyosarcoma	0-14	X	418	E-M-M	0	(0/1)[3]	n.s.
Glass et al., 1970	United States of America	1960-1966	0	Areas	s: same division<br>t: 1 year	Osteogenic sarcoma	0-14	X	620	E-M-M	0.111	(1/9)	Osteogenic, 0-14y, West north Central; Ewing, 0-14y, Pacific
						Ewings sarcoma	0-14	X	381	E-M-M	0.111	(1/9)	
Jensen et al., 1971	United States of America	1960-1967	0	Areas	s: same geographic division<br>t: 1 year	Retinoblastoma	0-14	X	269	E-M-M	0	(0/9)	n.s.
Morris, 1990	Worcestershire, Warwickshire, Staffordshire, Shropshire; UK	1953-1960	2	Geocodes	s: not specified<br>t: not specified	Nephroblastoma; Neuroblastoma	0-9	X	91	Knox	NA	(NA/NA)[1]	
Schneider et al., 1993	New Jersey, USA	1979-1985	2	Areas	s: same minor civil division (town)<br>t: 7, 14 months	Bone/soft tissue	0-14	X	5500[4]	E-M-M	0	(0/2)	n.s.
Gilman et al., 1995	England, Wales and Scotland, UK	1953-1980	3	Postcodes	s: 1, 2, 3, 4, 5, 10, 20 km<br>t: 0, 1, ..., 6, 9, 12, 18, 24, 48 months	Solid tumours	0-15	X	NA	Knox	NA	(NA/NA)[1]	

Table S4 continued

Author, Year	Study area	Period	Quality	Resolution	Critical lags	Cancer type	Age	Time	Cases	Method	Results		
											Rate of sig. Tests[a]		Significant test[b]
McNally et al., 2003	North West England, UK	1954-1998	3	Postcodes	s: Knox: 5 km, NN=b134/x136; K-f: 0.5-7.5 km, NN=127-141 t: Knox: 1 year; K-f: 1-15 months	Retinoblastoma	0-14	B	128	K-f	0	(0/2)	B: K-f: Wilms, 0-14y, NN; Wilms, >33 mths; Sarcomas, 0-14y; Rhabdomyosarcoma, 0-14y; Knox: Wilms, 0-14y; Wilms, >33 mths; Sarcomas, 0-14y X: n.s.
						Retinoblastoma	0-14	B	128	Knox	0	(0/2)	
						Neuroblastoma	0-14	B	287	K-f	0	(0/2)	
						Neuroblastoma	0-14	B	287	Knox	0	(0/2)	
						PNET	0-14	B	92	K-f	0	(0/2)	
						PNET	0-14	B	92	Knox	0	(0/2)	
						Wilms	0-14	B	229	K-f	0.5	(1/2)	
						Wilms	0-14	B	229	Knox	1	(2/2)	
						Wilms	<33 mths	B	NA	K-f	0	(0/2)	
						Wilms	<33 mths	B	NA	Knox	0	(0/2)	
						Wilms	>33 mths	B	NA	K-f	1	(2/2)	
						Wilms	>33 mths	B	NA	Knox	1	(2/2)	
						Hepatic	0-14	B	33	K-f	0	(0/2)	
						Hepatic	0-14	B	33	Knox	0	(0/2)	
						Sarcomas	0-14	B	370	K-f	1	(2/2)	
						Sarcomas	0-14	B	370	Knox	1	(2/2)	
						Osteosarcoma	0-14	B	105	K-f	0	(0/2)	
						Osteosarcoma	0-14	B	105	Knox	0	(0/2)	
						Rhabdomyosarcoma	0-14	B	159	K-f	1	(2/2)	
						Rhabdomyosarcoma	0-14	B	159	Knox	0	(0/2)	
						Germ cell	0-14	B	140	K-f	0	(0/2)	
						Germ cell	0-14	B	140	Knox	0	(0/2)	
						Retinoblastoma	0-14	X	128	K-f	0	(0/2)	

Table S4 continued

Author, Year	Study area	Period	Quality	Resolution	Critical lags	Cancer type	Age	Time	Cases	Method	Results		
											Rate of sig. Tests[a]		Significant test[b]
						Retinoblastoma	0-14	X	128	Knox	0	(0/2)	
						Neuroblastoma	0-14	X	287	K-f	0	(0/2)	
						Neuroblastoma	0-14	X	287	Knox	0	(0/2)	
						PNET	0-14	X	92	K-f	0	(0/2)	
						PNET	0-14	X	92	Knox	0	(0/2)	
						Wilms	0-14	X	229	K-f	0	(0/2)	
						Wilms	0-14	X	229	Knox	0	(0/2)	
						Hepatic	0-14	X	33	K-f	0	(0/2)	
						Hepatic	0-14	X	33	Knox	0	(0/2)	
						Sarcomas	0-14	X	370	K-f	0	(0/2)	
						Sarcomas	0-14	X	370	Knox	0	(0/2)	
						Osteosarcoma	0-14	X	105	K-f	0	(0/2)	
						Osteosarcoma	0-14	X	105	Knox	0	(0/2)	
						Rhabdomyosarcoma	0-14	X	159	K-f	0	(0/2)	
						Rhabdomyosarcoma	0-14	X	159	Knox	0	(0/2)	
						Germ cell	0-14	X	140	K-f	0	(0/2)	
						Germ cell	0-14	X	140	Knox	0	(0/2)	
McNally et al., 2006	Great Britain	1969-1993	3	Postcodes	s: K-f: NN=19-33, Knox: not specified<br>t: K-f: 0.1, 0.2, ..., 1.5 years; Knox: not specified	STS	0-14	X	2101	K-f	1	(1/1)	STS, 0-14y;<br>Osteosarcoma, 0-14y
						Bone tumors	0-14	X	1504	K-f	0	(0/1)	
						Osteosarcoma	0-14	X	811	K-f	1	(1/1)	
						Sympathetic nervous system tumours	0-14	X	2110	K-f	0	(0/1)	
						Wilms and other renal tumours	0-14	X	1889	K-f	0	(0/1)	
						Germ cell and related tumours	0-14	X	983	K-f	0	(0/1)	
						Germ cell and related tumours	0-14	X	983	K-f	0	(0/1)	

Table S4 continued

Author, Year	Study area	Period	Quality	Resolution	Critical lags	Cancer type	Age	Time	Cases	Method	Results	
											Rate of sig. Tests[a]	Significant test[b]
						Retinoblastoma,	0-14	X	941	K-f	0 (0/1)	
						Hepatic tumours	0-14	X	257	K-f	0 (0/1)	
						Carcinoma	0-14	X	877	K-f	0 (0/1)	
COMARE, 2006	England, Scotland and Wales, UK	1969-1993	3	Postcodes	s: 0.5, 1, 1.5, ..., 7.5 km<br>t: 0.1, 0.2, ..., 1.5 years	STS	0-14	X	2101	K-f	1 (1/1)	STS, 0-14y;<br>Osteosarcoma, 0-14y
						Bone tumors	0-14	X	1507	K-f	0 (0/1)	
						Wilms and other renal tumours	0-14	X	1890	K-f	0 (0/1)	
						Osteosarcoma	0-14	X	813	K-f	1 (1/1)	
McNally et al., 2009	Great Britain	1969-1993	3	Postcodes	s: K-f: NN=18-32, Knox: NN=25<br>t: K-f: 0.1, 0.2, ..., 1.5 years; Knox: 1 year	STS	0-14	B	1921	K-f	0 (0/1)	Osteosarcoma, 0-14y
						Bone tumors	0-14	B	1262	K-f	0 (0/1)	
						Osteosarcoma	0-14	B	669	K-f	1 (1/1)	
						Sympathetic nervous system tumours	0-14	B	2002	K-f	0 (0/1)	
						Wilms and other renal tumours	0-14	B	1820	K-f	0 (0/1)	
						Germ cell and related tumours	0-14	B	915	K-f	0 (0/1)	
						Retinoblastoma,	0-14	B	911	K-f	0 (0/1)	
						Hepatic tumours	0-14	B	245	K-f	0 (0/1)	
						Carcinoma	0-14	B	789	K-f	0 (0/1)	
Amin et al., 2010	Florida, USA	2000-2007	3	Areas	s: not specified<br>t: individual years (not specified)	other	0-19	X	4591	Scan	NA (NA/NA)[1]	
Ortega-Garcia et al., 2011	Murcia, Spain	1998-2009	2	Areas	s: BHA<br>t: months (exact unit unclear)	SNS	0-14	X	29	Scan	1 (1/1)	SNS, 0-14y, 3 BHA, 07/01-01/07
						Retinoblastoma	0-14	X	5	Scan	0 (0/1)	
Ortega-Garcia et al., 2015	Murcia, Spain	1998-2013	2	Areas	s: census tracts<br>t: months	SNS	0-14	X	17	Scan	0 (0/1)	n.s.

Table S4 continued

Author, Year	Study area	Period	Quality	Resolution	Critical lags	Cancer type	Age	Time	Cases	Method	Results	
											Rate of sig. Tests[a]	Significant test[b]
Kreis et al., 2016	Switzerland	1985-2010	4	Geocodes	s: 0.5, 1, 2, 5, 10 km<br>t: 6, 12, 18, 24 months	Neuroblastomas	0-15	B	268	Knox	0 (0/1)	B: Neuroblastomas, 0-15y, 10km, 2y; STS, 0-15y, 5km, 2y<br>X: n.s.
						Neuroblastomas	0-15	X	310	Knox	0 (0/1)	
						STS	0-15	B	229	Knox	0 (0/1)	
						STS	0-15	X	334	Knox	0 (0/1)	

[a]Proportion of significant space-time clustering tests per analysis group (number of clustering tests signficant at 5% alpha-level/total number of clustering tests performed).

[b]Characteristics of (most) significant space-time clustering test, specifying (if applicable): time frame of birth B or diagnosis X; clustering test: diagnostic group, age range, spatial lag, temporal lag, sub-area and sub-period. Reported results are unadjusted for multiple testing.

Critical lags: *s* spatial lag, *t* temporal lag, *NN* nearest neighbors, *M* Mantel test, *K-f* K-functions, *K* Kulldorff's scan statistic, *BHA* Basic Health Area; **Cancer type:** *PNET* peripheral neuroectodermal tumours, *SNS* sympathetic nervous system tumour, *STS* soft tissue sarcomas; **Time:** *B* date of birth, *X* date of diagnosis; **Method:** *E-M-M* Ederer-Myers-Mantel, *K-f* K-functions, *Scan* Kulldorff's scan statistic; **Results:** *NA* not applicable, *n.s.* not significant, *M* Mantel test, *K-f* K-functions, *K* Kulldorff's scan statistic, *m* mile(s), *d* days, *mth(s)* month(s), *y* year(s).

[1]Excluded as other tumours were not analzed as separate diagnostic group

[2]Summation across states

[3]Summation across divisions

[4]All diagnostic groups
