# Supplementary material for: Space–time clustering of childhood cancers: a systematic review and pooled analysis
Source: Eur J Epidemiol. 2018 Nov 16;34(1):9–21. doi: 10.1007/s10654-018-0456-y (PMC6326085; doi:10.1007/s10654-018-0456-y)
Supplement: Supplementary file 7 — Supplementary material 7 (PDF 90 kb) [file 10654_2018_456_MOESM7_ESM.pdf]

Table S5 Quality criteria of space-time clustering studies

Author, Year	Source of case ascertainment	Spatial resolution	Multiple Testing	Population Shifts	Score
Pinkel et al., 1959	Cancer registry of Erie County and New York State Health Departments	Street address			2
Pinkel et al., 1963	Cancer registry of the State of New York; local hospital records	Street address			2
Ederer et al., 1964	Tumor Registry, Connecticut State Department of Health	169 towns			1
Knox, 1964	Regional Cancer Registration Bureau; hospital records; death certificates	National grid (0.5km)			1
Meighan et al., 1965	Death certificates[1]	Geocodes (0.1km)			1
Ederer et al., 1965	Connecticut Tumor Registry	169 town units		5-year study periods	2
Fraumeni et al., 1966	Bureau of Cancer Control, New York State Health Department; death certificates	417 cities and towns			1
Mainwaring, 1966	Alder Hey and Royal Liverpool Children's Hospitals	Geocodes (0.1km); 40 electoral wards			1
Pike et al., 1967	Angal Mission, Arua Government and Kuluva Mission Hospitals	Geocodes			1
Stark et al., 1967	Death certificates from the Michigan State Department of Health	State; 83 counties			0
Till et al., 1967	Oxford Survey of Childhood Malignancies; Hospital records; Death certificates	Geocodes (0.1km)			2
Gunz et al., 1968	New Zealand Department of Health Cancer Death Lists	Postcodes (1 mile)			1
Browning et al., 1968	23 hospitals; Department of Vital Statistics Ohio	not specified; 34 municipalities			0
Glass et al., 1968	Death certificates from the National Center for Health Statistics	Geocodes (150m); 32 census regions			1
Klauber, 1968	Death and birth certificates; California Tumor Registry; hospital records	468 hospitals			1
Miller et al., 1968	Death certificates from the National Vital Statistics, Public Health Service	51 States; 9 standard divisions			0
Williams et al., 1969	Kuluva and Arua Hospital	Geocodes			1
Glass et al., 1969	National Center for Health Statistics	Address (200m)			1
Fraumeni et al., 1969	Death certificates from the National Vital Statistics, Public Health Service	51 states; 9 stand. divisions; 4 regions			0
Li et al., 1969	Death certificates from the National Vital Statistics, Public Health Service	9 standard divisions			0
Klauber et al., 1970	San Francisco Department of Public Health; Medical Society; Stanford University	Geocodes		4 separate 5-year periods	2
Bailar et al., 1970	Hospital records; death certificates	169 administrative areas (towns)			0
Glass et al., 1970	Death certificates[2]	9 geographic divisions			0
Zahálková et al., 1970	Paediatric University Clinics, district hospitals and paediatricians	13 districts			0
Glass et al., 1971	Cancer Death List; New Zealand Department of Health	Geocodes		2 consecutive 6-year periods	2
Jensen et al., 1971	US Public Health Service death certificates	9 geographic divisions			0
Brubaker et al., 1973	Shirati Hospital Registry; Kampala Cancer Registry	Geocodes			2
Evatt et al., 1973	Leukemia registry of metropolitan Atlanta area	111 census tract units		growth curve modelling	2

Table S5 continued

Author, Year	Source of case ascertainment	Spatial resolution	Multiple Testing	Population Shifts	Score
Larsen et al., 1973	Ongoing population-based leukemia/lymphoma registry	18 census tracts		n/a[3]	2
Smith et al., 1976	Oxford Survey of Childhood Malignancies; Hospital records; Death certificates	Geocodes	not specified		3
Morrow et al., 1976	Kampala Cancer Registry	Geocodes			2
Morrow et al., 1977	Kampala Cancer Registry	Geocodes (1km)			2
Williams et al., 1978	Hospital records; Lymphoma Treatment Centre Uganda Cancer Institute; Uganda Cancer Registry	Geocodes; 10 administrative districts			2
Biggar et al., 1979	Burkitt's Tumor Project at Korle Bu Hospital in Accra	Home community			0
Siemiatycki et al., 1980	Shirati Hospital Register (North Mara)	Geocodes			1
Van Steensel-Moll et al., 1983	Morbidity Registry of the Dutch Childhood Leukaemia Study Group	<500m (approx.)[4]			1
Pinder, 1985	Mersey Regional Cancer Registry	Postcodes			2
Mangoud et al., 1985	North Western Regional Cancer Registry	Postcodes (100m)			2
Morris, 1990	Oxford Survey of Childhood Cancers; Regional Cancer Registry Birmingham	Geocodes (100m)			2
Gilman et al., 1991	National Registry of Childhood Tumours	Postcode; grid reference		Klauber and Mustacchi (1970)	3
Knox et al., 1992	National Registry of Childhood Tumours	Postcodes (100m)		moving time window of 1000 days	3
Van Den Bosch et al., 1993	Burkitt's Lymphoma Research Unit at Kamuzu Central Hospital	Geocodes (200m)			1
Schneider et al., 1993	New Jersey Cancer Registry	567 Minor Civil Divisions (towns)		analysis for 3 time periods of 28 months	2
Gilman et al., 1995	Oxford Survey of Childhood Cancers	Postcodes (100m)		Klauber and Mustacchi 1970	3
Petridou et al., 1996	Hospital Archives; National Network of Childhood Oncologists	Geocodes			2
Alexander et al., 1998	Specialist Cancer Registries	Small census areas			1
Gustafsson et al., 1999	Swedish Cancer Registry; Swedish Medical Birth Register	277 Municipalities	Bonferroni		2
Gilman et al., 1999	Leukemia Research Fund Data Collection Survey	Postcodes (100m)	Rodrigues et al. 1992		3
Hjalmars et al., 1999	Swedish Cancer Registry	2507 parishes	Baker's Max/ Scan statistics	Kulldorff & Hjalmars 1999	3
Gilman et al., 1999	Leukaemia Research Fund Data Collection Study	Geocodes	Rodrigues et al. 1992		3
Birch et al., 2000	Manchester Children's Tumour Registry	Geocodes 1954-73; Postcodes (100m) 74-85	K-function		3
Gustafsson et al., 2000	Swedish Cancer Registry; Swedish Medical Birth Registry	277 municipalities	Bonferroni		2
McNally et al., 2002	Manchester Children's Tumour Registry	Postcodes (100m)	K-function		3
McNally et al., 2002	Manchester Children's Tumour Registry	Postcodes (100m)	K-function		3
McNally et al., 2003	Manchester Children's Tumour Registry	Postcodes (100m)	K-function		3
McNally et al., 2004	Manchester Children's Tumour Registry	Postcodes (100m)	K-function		3

Table S5 continued

Author, Year	Source of case ascertainment	Spatial resolution	Multiple Testing	Population Shifts	Score
Houben et al., 2005	Eindhoven Cancer Registry	Postcodes (nbhds/small municipalities)	K-function		3
Akhtar et al., 2005	Two Tertiary Care Hospitals in Karachi	Geocodes (1km)			1
McNally et al., 2006	National Registry of Childhood Tumours	Postcodes (100m)	K-function		3
Bellec et al., 2006	French National Registry of Childhood Haematopoietic Malignancies	3644 Cantons; 36343 Communes	Scan statistics	interpolation between 1990/1999 census	3
COMARE, 2006	National Registry of Childhood Tumours	Postcodes (100m)	K-function		3
Kearney, 2008	Florida Cancer Data System	3154 Census Tracts	Scan statistics	linear interpolation	3
McNally et al., 2009	National Registry of Childhood Tumours	Postcodes (100m)	K-function		3
Schmiedel et al., 2010	German Childhood Cancer Registry	Municipalities; 30x30km squares	Scan statistics		2
Amin et al., 2010	Florida Association of Pediatric Tumor Programs	972 Zip code areas	Scan statistics	n/a[5]	3
Ortega-Garcia et al., 2011	Registry of the Virgen de la Arrixaca Hospital	85 Basic Health Areas	Scan statistics	Directly Standardized Rates	2
Demoury et al., 2012	French National Registry of Childhood Haematopoietic Malignancies	1895 Living Zones[6]	Scan statistics		2
McNally et al., 2012	Yorkshire Specialist Register of Cancer in Children and Young People	Postcodes (100m)	K-function/ Scan statistics		3
Ortega-Garcia et al., 2015	Provincial Cancer Registry (MACAPEMUR)	386 Census tracts	Scan statistics		2
Kreis et al., 2016	Swiss Childhood Cancer Registry	Geocodes	Baker's Max	Kulldorff and Hjalmars 1999	4

[1]Source unknown

[2]1960-64 all USA; 1965-66 USA excluding Louisiana and Missouri

[3]Effect excluded by comparison with cases aged 15-54

[4]Addresses were located on 1:50,000 scale map and larger scale street maps in larger towns

[5]Analysis of 55 large counties instead of ZIP code areas made little difference

[6]Mean population per Living Zone 30,500 people
