# Supplementary material for: Space–time clustering of childhood cancers: a systematic review and pooled analysis
Source: Eur J Epidemiol. 2018 Nov 16;34(1):9–21. doi: 10.1007/s10654-018-0456-y (PMC6326085; doi:10.1007/s10654-018-0456-y)
Supplement: Supplementary file 8 — Supplementary material 8 (PDF 85 kb) [file 10654_2018_456_MOESM8_ESM.pdf]

Table S6 Quantitative synthesis of results of space-time clustering analyses of childhood leukaemia for time of diagnosis and birth: Number of studies individually reporting significant evidence of space-time clustering and mean proportion of significant tests (MPST) for different study data and methodological characteristics*

Category	Diagnosis		Birth	
	n[a]/No[b]	MPST[c]	n[a]/No[b]	MPST[c]
Leukaemia	10/23	26%	1/9	11%
Study Period				
Begins in 1980 or later	2/6	15%	1/2	50%
Ends in 1990 or later	1/6	17%	1/4	25%
Study region				
UK	3/7	28%	0/5	0%
USA	4/8	24%	0/2	0%
Europe	2/6	22%	1/2	50%
Sample size				
N > 1000	3/8	32%	1/3	33%
N < 1000	9/22	17%	0/7	0%
Geocoding				
Geocodes	5/10	26%	1/4	25%
Postcodes	1/6	17%	0/2	0%
Areas	4/10	23%	0/3	0%
Population shifts adjusted	3/8	20%	1/1	100%
Methods score ≥ 3	2/6	30%	1/5	20%
Clustering test				
Knox	7/19	14%	1/6	17%
Mantel	1/5	19%	0/1	0%
K-functions	2/3	67%	0/3	0%
Kulldorff	2/5	40%	0/0	

[a]Number of studies individually reporting significant evidence of clustering (proportion of significant clustering tests > 0.05)

[b]Number of studies included (excluding studies with overlapping samples and five studies employing scan statistics as clustering test listed separately under the Kulldorff category)

[c]Mean proportion of significant clustering tests across included studies

*Total number of studies might vary by stratified analysis because of differing exclusions due to overlapping study samples (cf. Section "Excluded studies" in the online supplementary material)
